# Supplementary material for: A scalable, open-source implementation of a large-scale mechanistic model for single cell proliferation and death signaling
Source: Nat Commun. 2022 Jun 21;13:3555. doi: 10.1038/s41467-022-31138-1 (PMC9213456; doi:10.1038/s41467-022-31138-1)
Supplement: Supplementary file 1 — Supplementary Information [file 41467_2022_31138_MOESM1_ESM.pdf]

SUPPLEMENTARY FIGURES

A Scalable, Open-Source Implementation of a Large-Scale Mechanistic Model for Single Cell Proliferation and Death Signaling

Cemal Erdem, Arnab Mutsuddy, Ethan M. Bensman, William B. Dodd, Michael M. Saint-Antoine, Mehdi Bouhaddou, Robert C. Blake, Sean M. Gross, Laura M. Heiser, F. Alex Feltus, and Marc R. Birtwistle

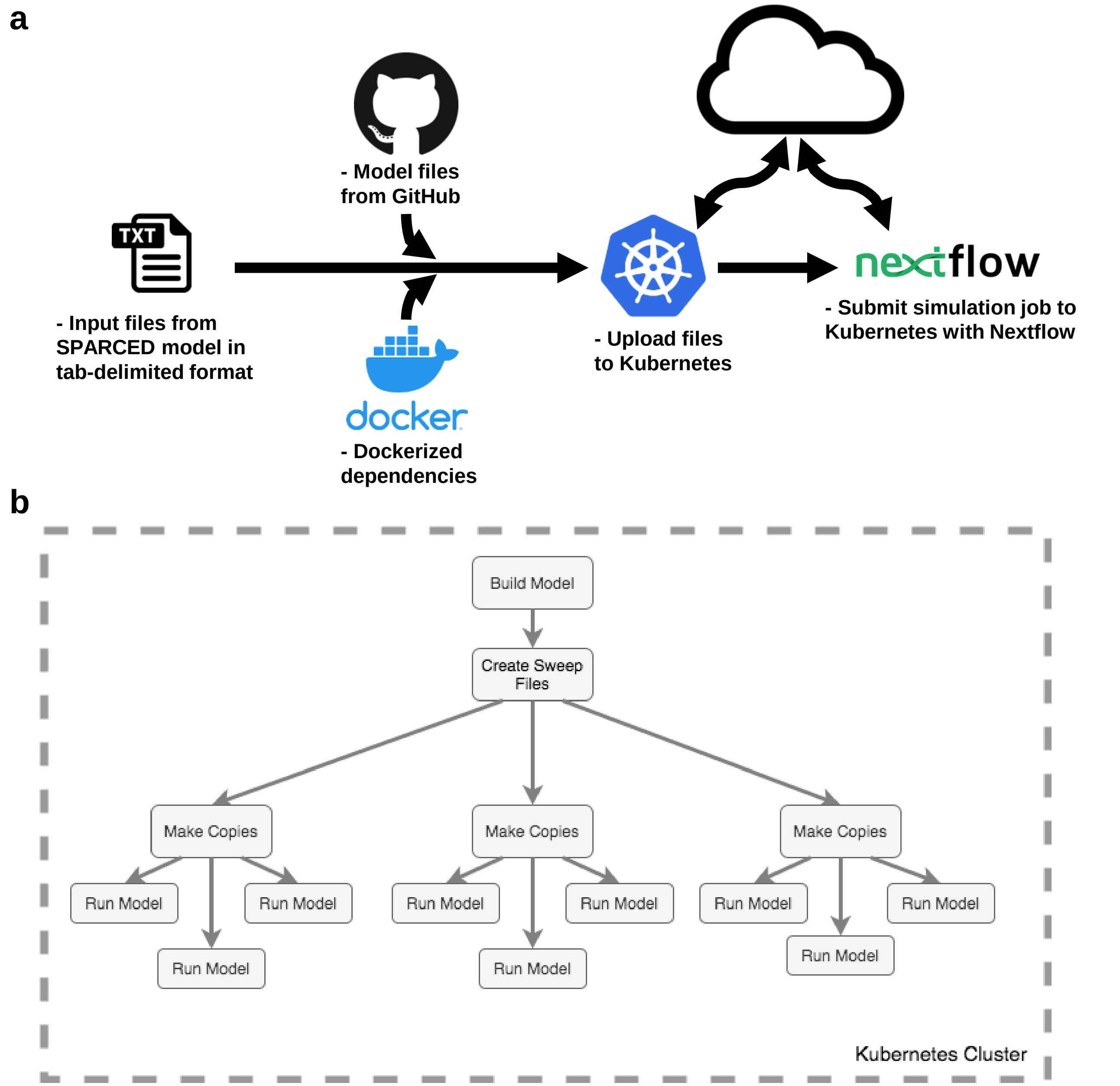

Supplementary Fig. 1 SPARCED-nextflow is cloud-computing ready version of the SPARCED pipeline. a SPARCED-nf implementation overview. **b** Details of SPARCED-nf pipeline, where users can run multiple instances of SPARCED models in parallel, each with different parameter values set by the config file.

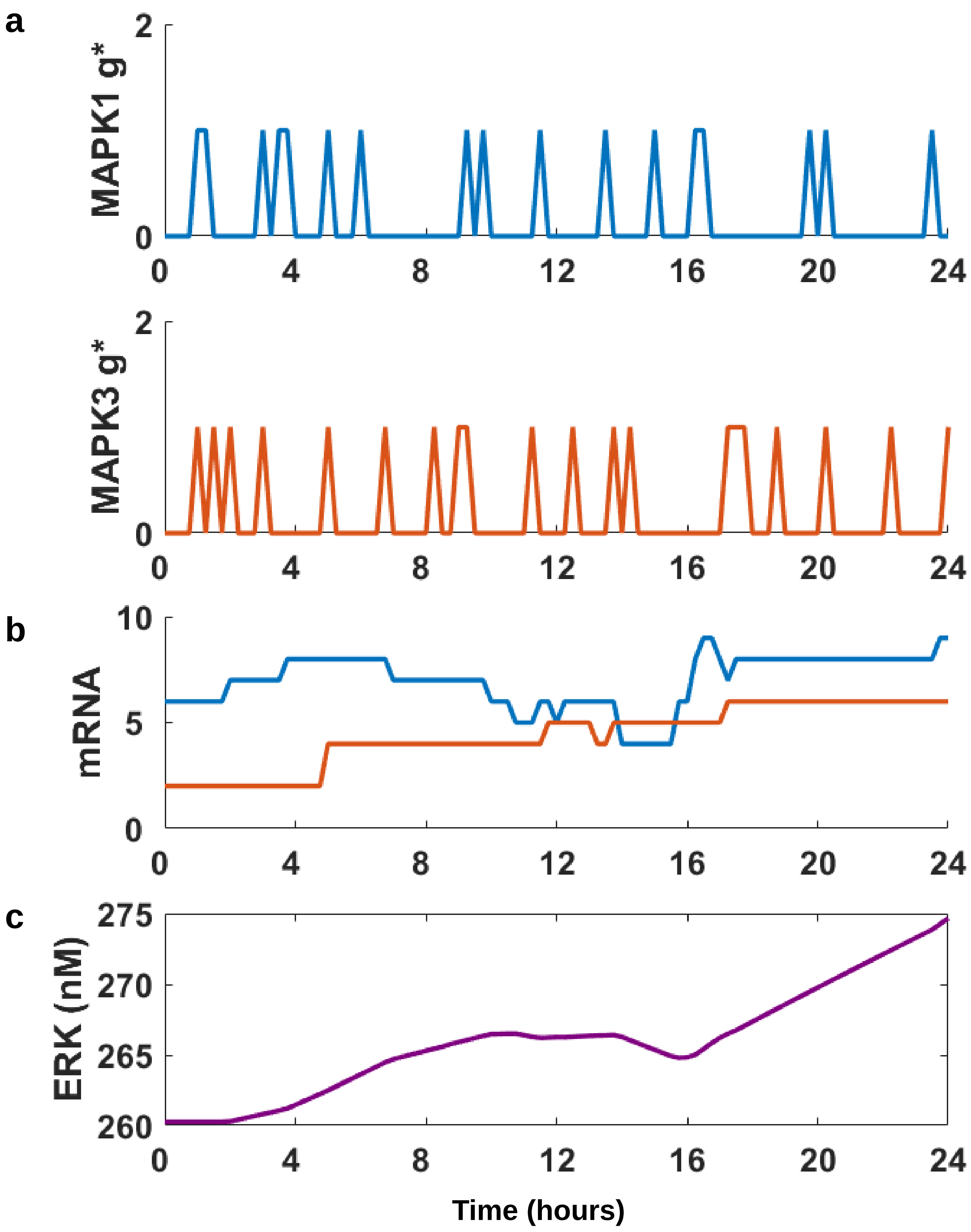

Supplementary Fig. 2 SPARCED model includes a stochastic gene expression module. a Two isoforms of ERK gene (MAPK1 and MAPK3) are activated randomly. **b** It leads to two distinct mRNAs species. **c** The ERK1 and ERK2 mRNAs are translated into a single ERK protein. The trajectories are obtained from a stochastic single cell simulation with no growth factor stimulation for 24 hours. Source data are provided in Source Data.

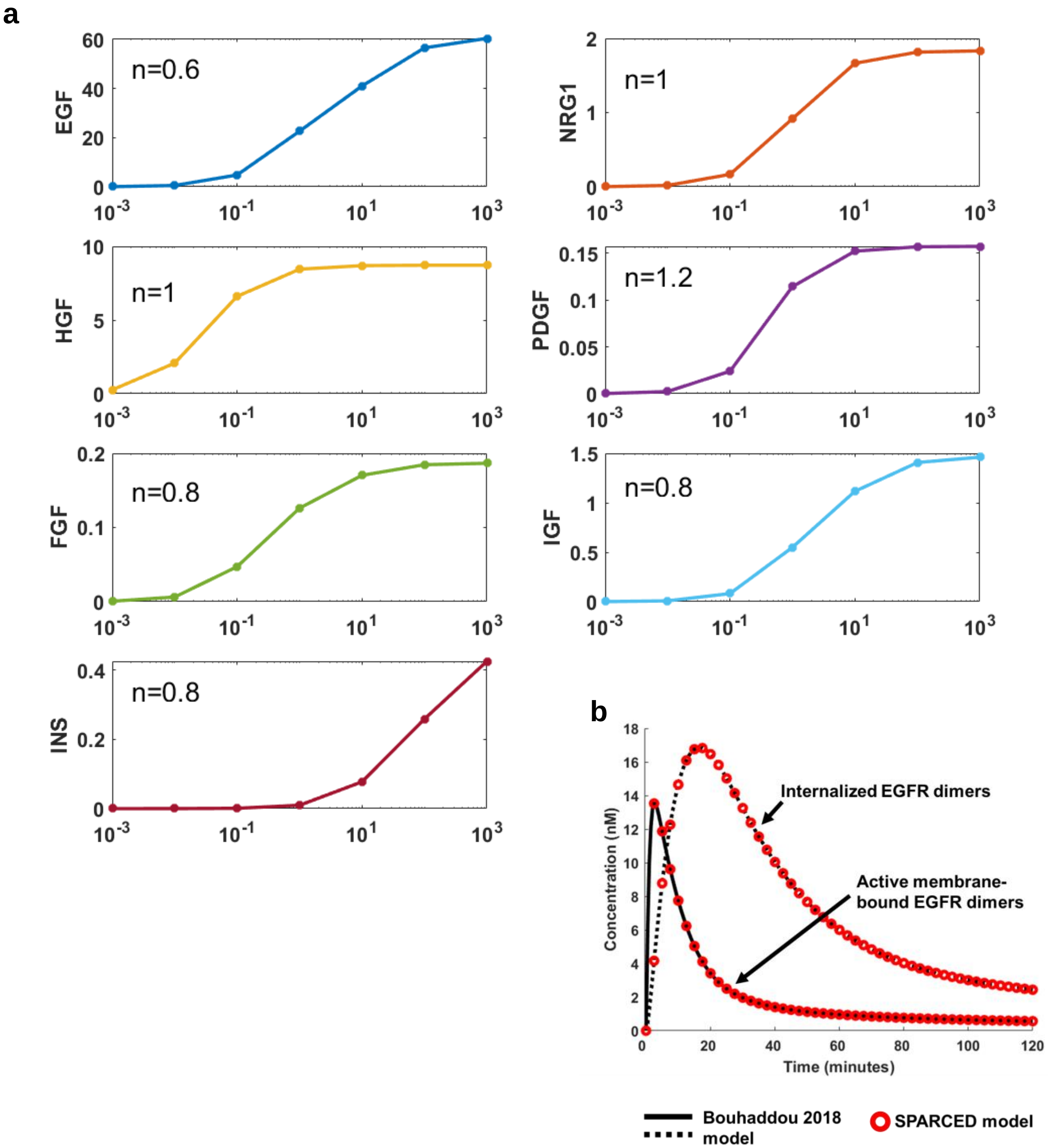

Supplementary Fig. 3. The SPARCED model recapitulates ligand-receptor cooperativity observations. a Hill coefficients for each ligand-receptor pair in MCF10A context. The simulations capture literature knowledge. **b** The dynamics of activated EGFR (membrane-bound and internalized) dimers are recaptured by the SPARCED model, compared to Bouhaddou2018 model. Source data are provided in Source Data.

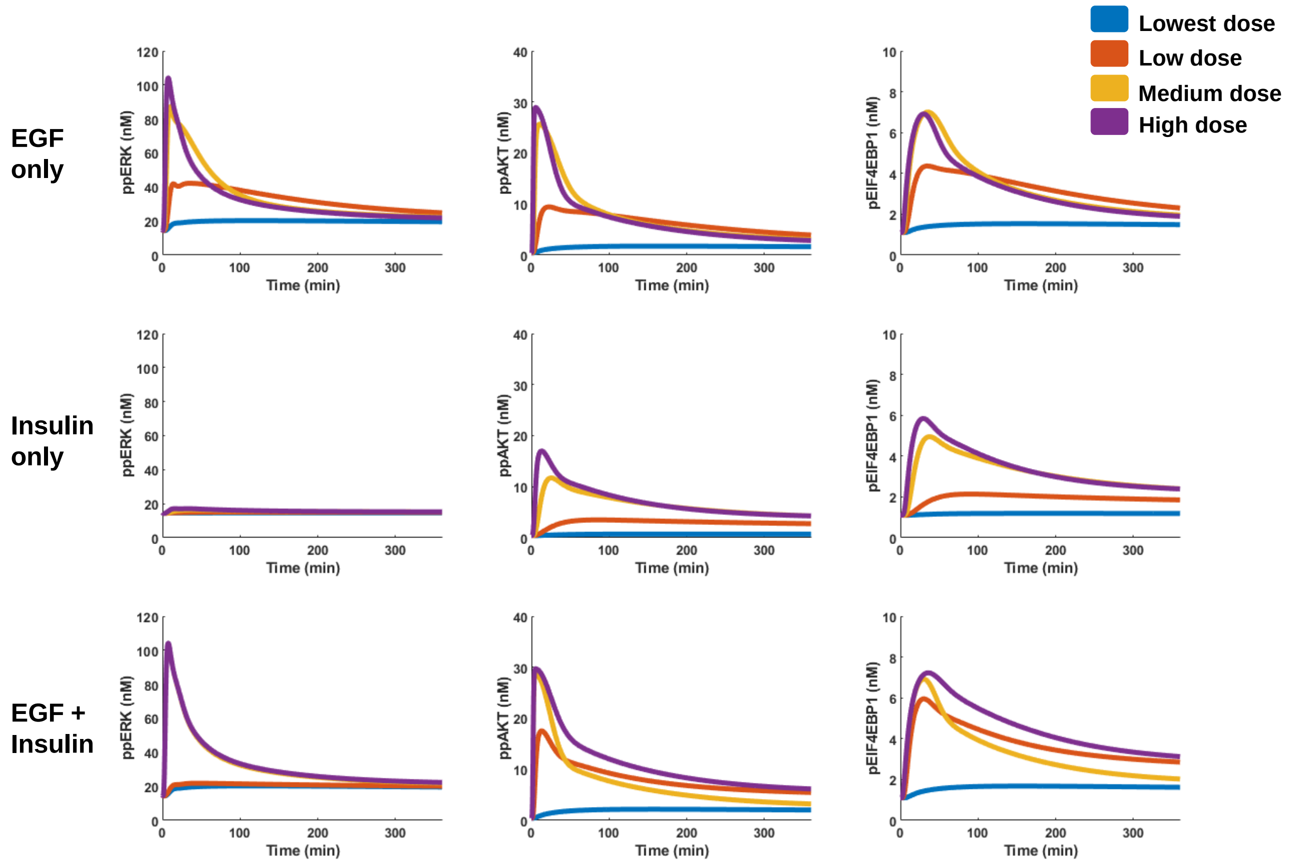

Supplementary Fig. 4 Model response to EGF and insulin. Signaling dynamics of ppERK, ppAKT, and pEIF4EBP1 induced by EGF, Insulin, or EGF+Insulin treatment for 6 hours. Serum-starved MCF10A cells are stimulated with EGF (0.01, 0.1, 1, and 10 nM), Insulin (0.17, 1.7, 17, and 1721 nM), or EGF+Insulin (0.01+0.17, 10+0.17, 0.01+1721, and 10+1721 nM). Source data are provided in Source Data.

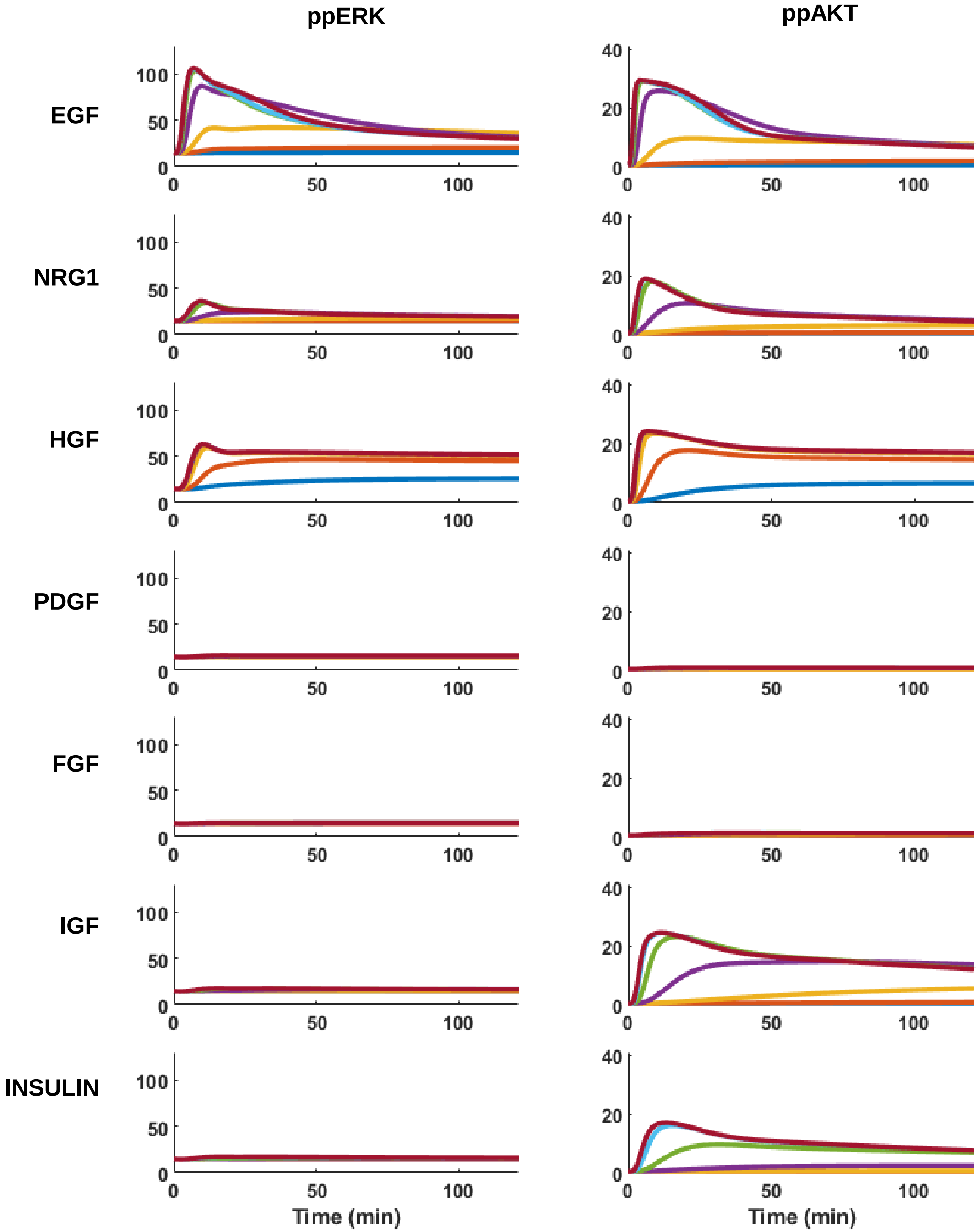

Supplementary Fig. 5 Each ligand induces different ERK and AKT responses. Signaling dynamics of ppERK and ppAKT induced by EGF, Heregulin (NRG1), HGF, PDGF, FGF, IGF, and Insulin treatment for 2 hours. Serum-starved MCF10A cells are stimulated with corresponding ligands at a dose range of 0.001 to 1000 nM. Source data are provided in Source Data.

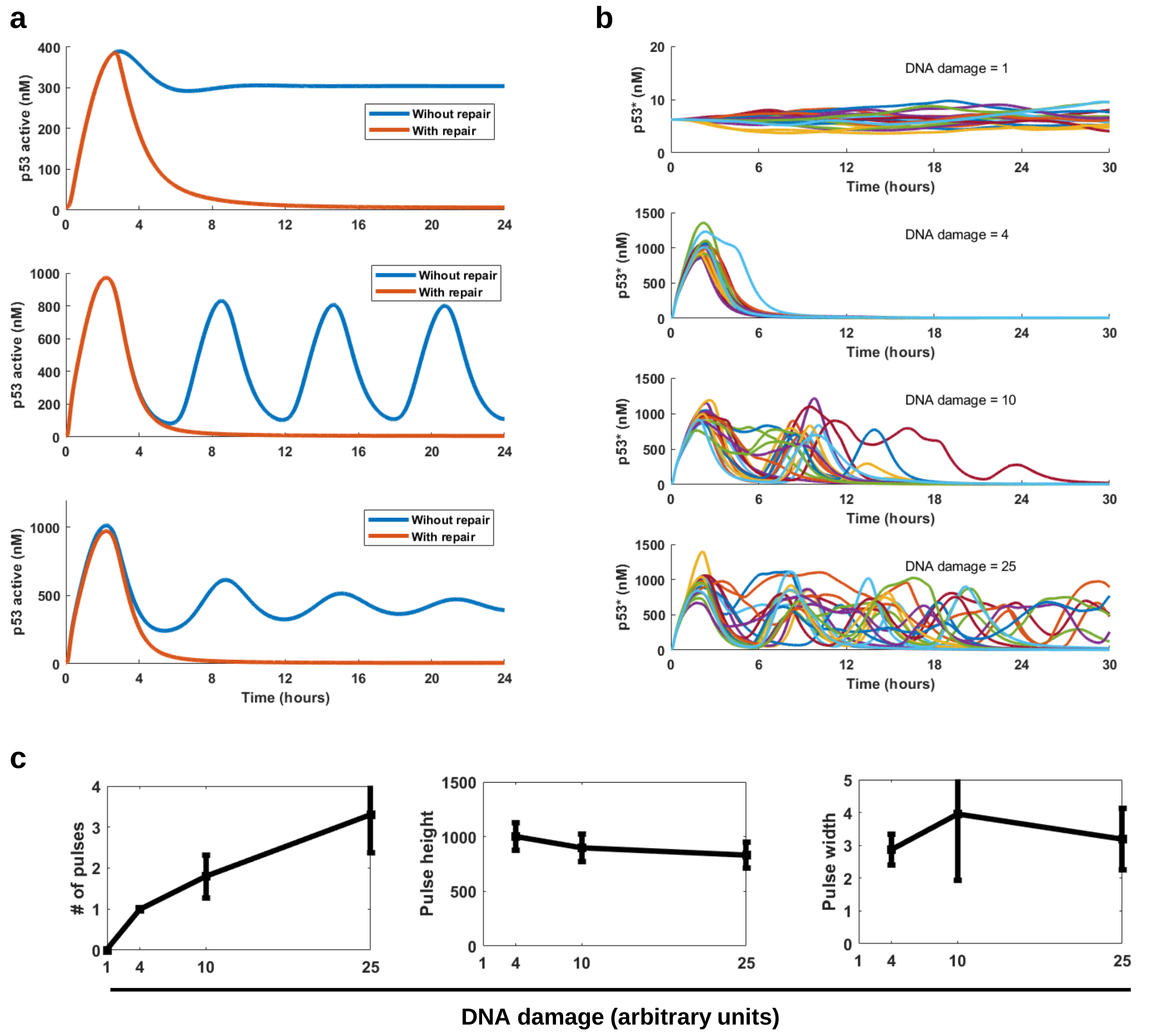

Supplementary Fig. 6 SPARCED includes a DNA damage and DNA repair submodels. a p53 is activated in response to double (middle) or/and single (top/bottom) stranded DNA break damage. When DNA break repair mechanism is turned on (orange curves), p53 activity (or oscillatory behavior) dies down. **b** Single cells show different levels of p53 response to DNA damage. Increasing DNA damage amount (top to bottom) leads to higher number of activated p53 peaks. **c** The number of p53 pulses increases with increasing DNA damage, whereas pulse height and width remain relatively constant (results based on simulations shown in **(b)**). Plots show mean ± s.e.m. for N=20 starting cell simulations. Source data are provided in Source Data.

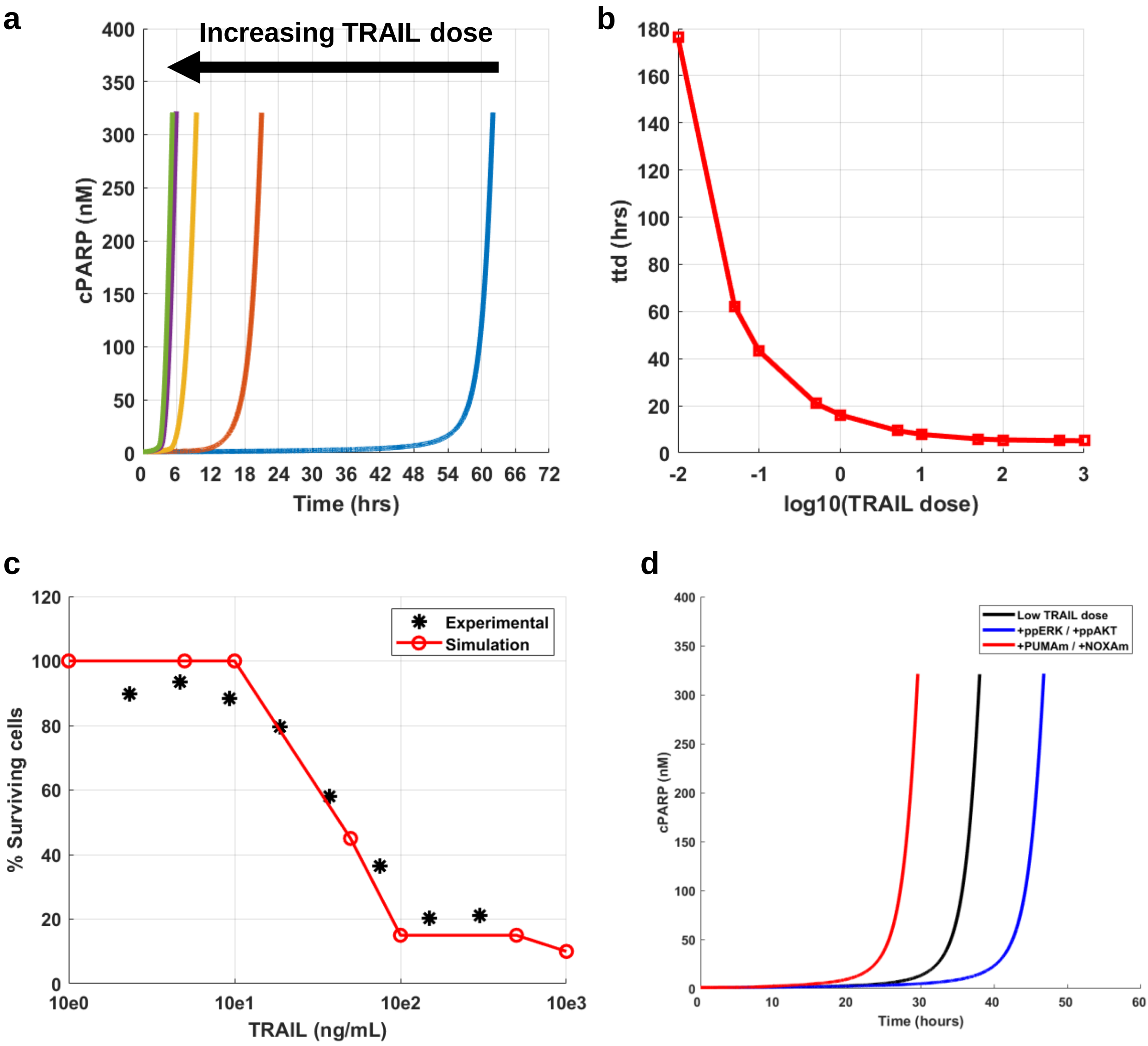

Supplementary Fig. 7 SPARCED includes a functional cell death submodel. a Increasing TRAIL dose decreases the time it takes to die (ttd) for the average cell. Representative cells trajectories are shown, where the cells are simulated deterministically with different doses of TRAIL until they die (or up to 100 hours). The time of death is defined by the amount of cleaved PARP (cPARP, y-axis) surpassing the amount un-cleaved PARP. **b** Summary of ttd values for different TRAIL doses. **c** The fraction of surviving cells decreases as stimulated TRAIL dose increases. The red circles represent percentage of living cells when 20 stochastic single cells are simulated with specified TRAIL dosage for 5 hours. The black stars are experimental data from Bouhaddou 2018 model. **d** Increasing ERK and AKT activity levels prolongs TRAIL induced time to death (blue curve), whereas increasing PUMA and NOXA expression levels decreases the time it takes for cells to die (red curve). Cells with specified alterations are compared to the cell stimulated with a low dose of TRAIL (black curve). cPARP levels are the proxy for cell death, where the cells go apoptosis when [cPARP]>[PARP]. Source data are provided in Source Data.

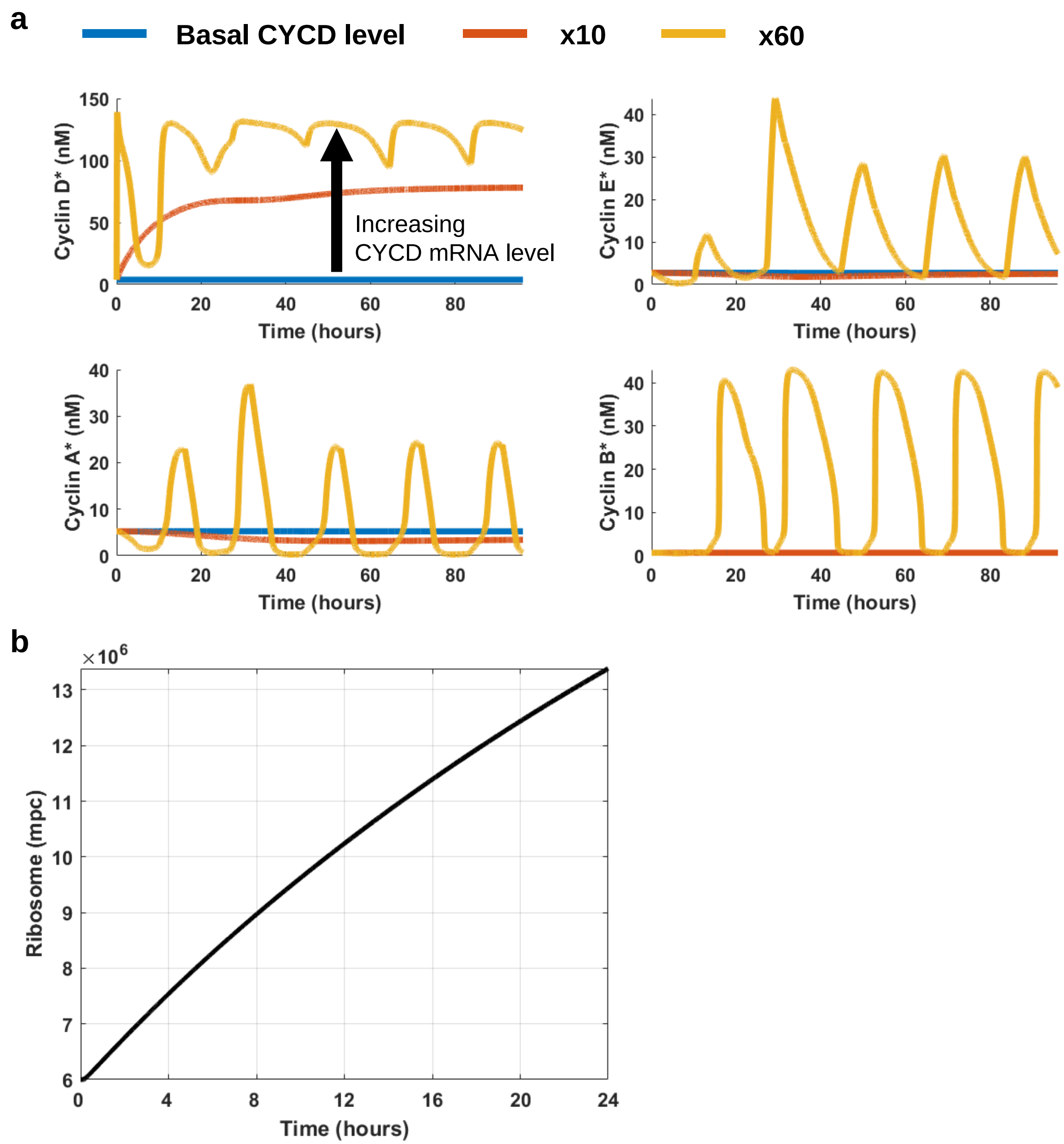

Supplementary Fig. 8 The cell cycle submodel of SPARCED is functional and the ribosome number increases as the cell progresses. a Cyclin D mRNA levels induces proper cyclin-CDK complex progression and oscillations for cell cycle entry. Plots show Cyclin D, E, A, and B concentrations when basal (blue), 10X basal (dark orange), and 60X basal (light orange) levels of Cyclin D mRNA (CYCD) are simulated. **b** The number of ribosomes in the cell doubles around 20 hours. The cell is simulated with full growth condition (EGF=100 nM, NRG1=100 nM, HGF=100 nM, PDGF=100 nM, FGF=100 nM, IGF=100 nM, INS=100 nM). Source data are provided in Source Data.

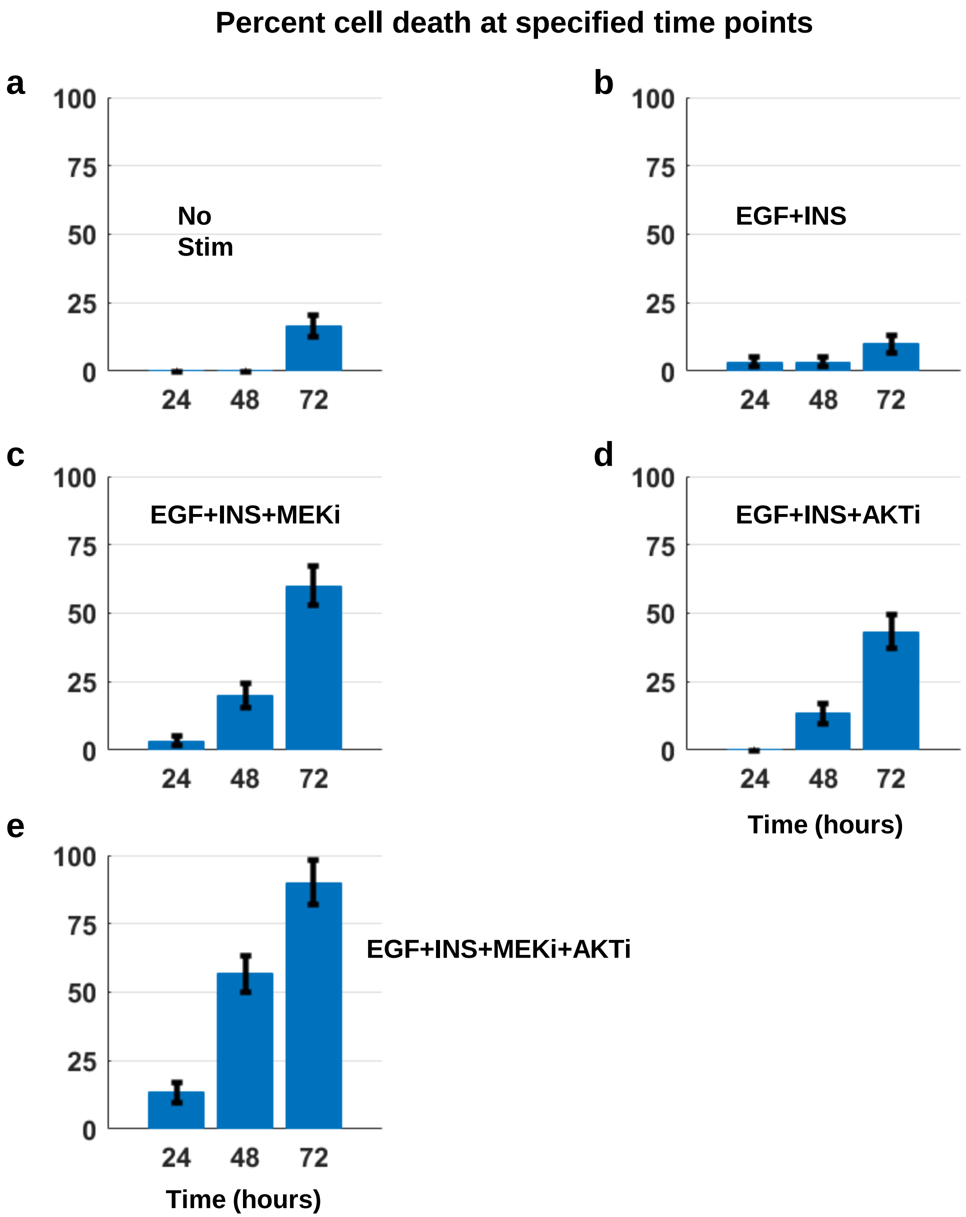

Supplementary Fig. 9 Inhibition of AKT and ERK pathways together synergistically increase cell death, in EGF and insulin stimulated cells. Serum-starved MCF10A cells are simulated with following conditions: **a** No stimulation, **b** EGF=20ng/mL + Insulin=10μg/mL, **c** EGF=20ng/mL + Insulin=10μg/mL + MEKi=10μM, **d** EGF=20ng/mL + Insulin=10μg/mL + AKTi=10μM, and **e** EGF=20ng/mL + Insulin=10μg/mL + MEKi=10μM + AKTi=10μM for up to 80 hours. **(a-e)** The bar plots show mean ± s.e.m. of time to death (ttd) for N=30 starting cells. The ttd are captured by cPARP spikes. Source data are provided in Source Data.

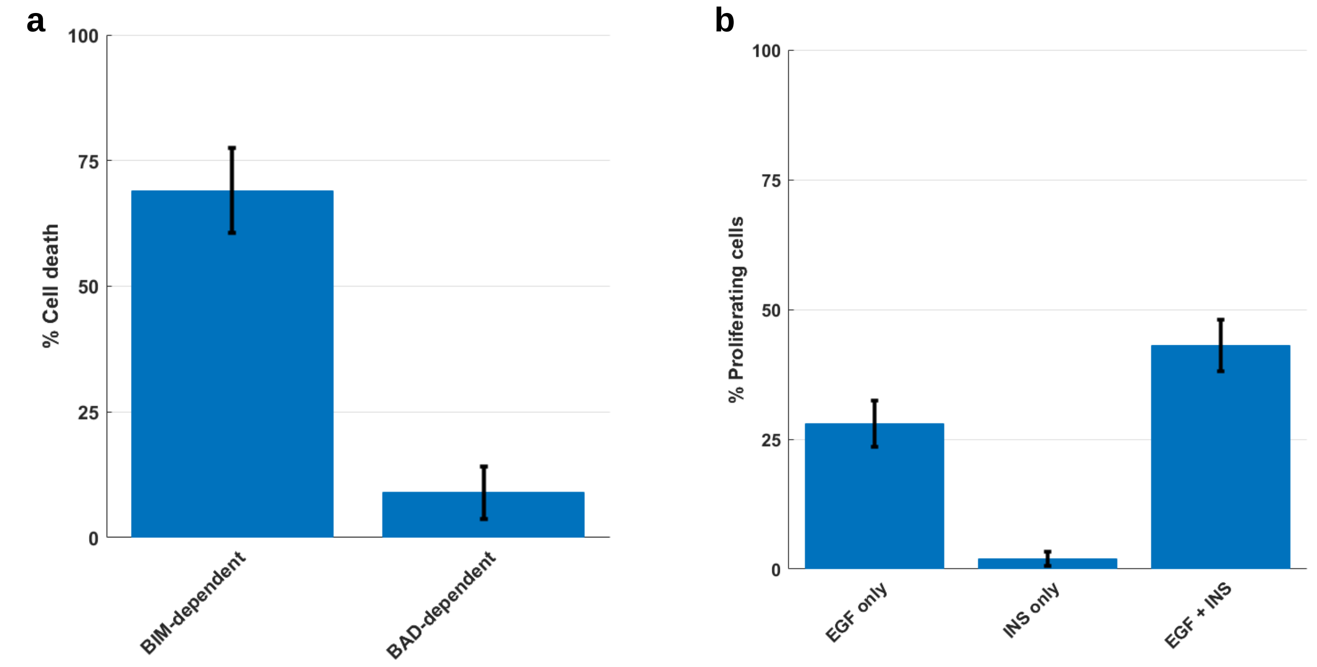

Supplementary Fig. 10 BIM dependent cell death in response to ERK and AKT inhibition. a Simulations where BIM-dependent or BAD-dependent mechanisms are switched off and percent death calculated in response to EGF + insulin at 48 hours. The results show that ERK and AKT inhibition induced cell death mechanisms are mostly BIM dependent, not BAD. Bars represent mean percent cell death ± s.e.m for N=100 stochastic cell simulations. **b** EGF and insulin cooperatively induce cell cycle entry, with insulin inducing very little cell cycle entry alone. Cells are simulated with EGF (10nM), Insulin (1721nM), or EGF+Insulin (10nM+1721nM) for 30 hours and the percentage of cells entering S-phase are calculated. Cells are considered in S-phase when the sum of concentrations of Cyclin E, A, and B is greater than 20nM. Bars represent percent cell death ± s.e.m. for N=100 stochastic cell simulations. Source data are provided in Source Data.

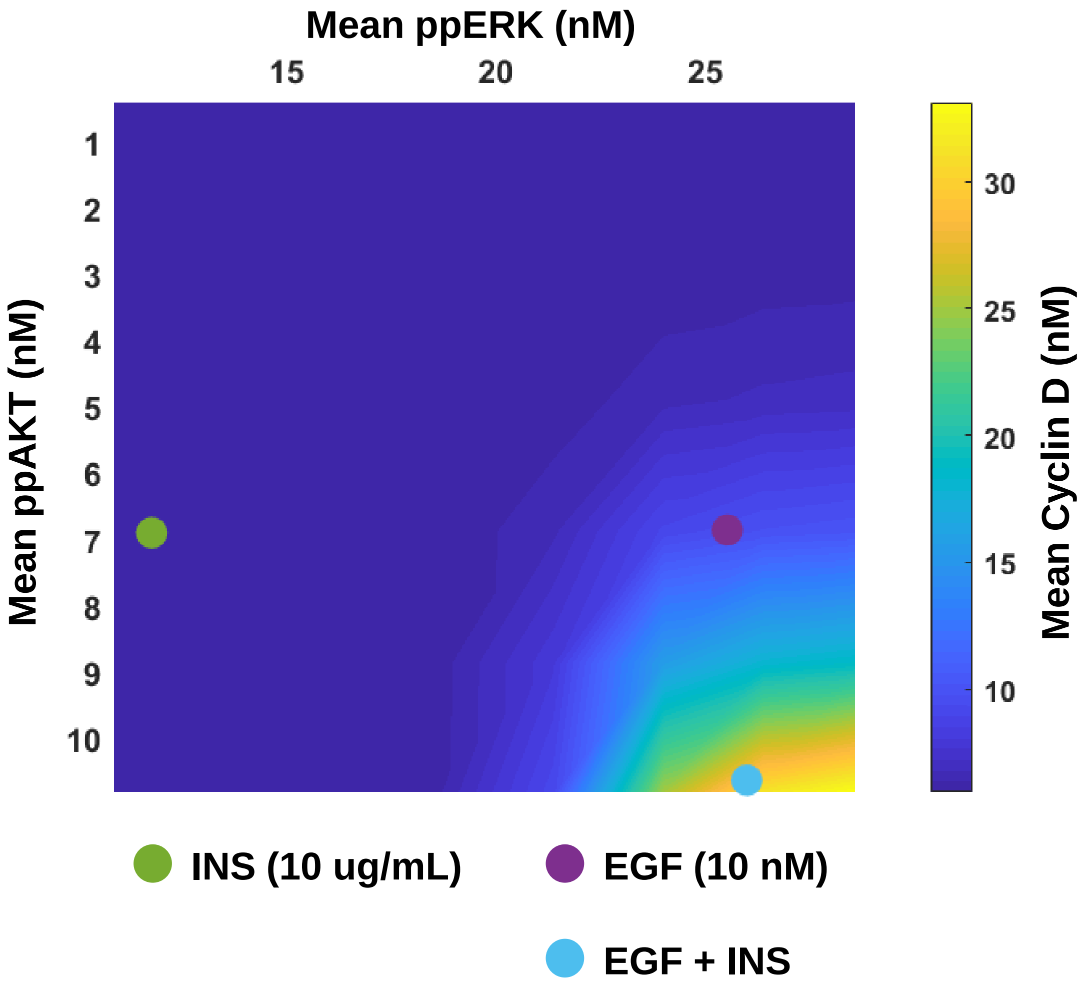

Supplementary Fig. 11 Cell cycle entry requires activation of both AKT and ERK pathways. Activation of both ERK and AKT pathways are required for robust cell cycle entry. Time averaged ppERK and ppAKT levels correlate with Cyclin D levels. Basal levels of ppERK and ppAKT are increased (between 1X-20X) and each condition is simulated up to 6 hours. The time-averaged levels of ppERK and ppAKT are plotted against the time-averaged Cyclin D levels. Conditions representing EGF (10nM), Insulin (1721nM), and EGF+Insulin (10nM+1721nM) are shown with colored circles. Source data are provided in Source Data.

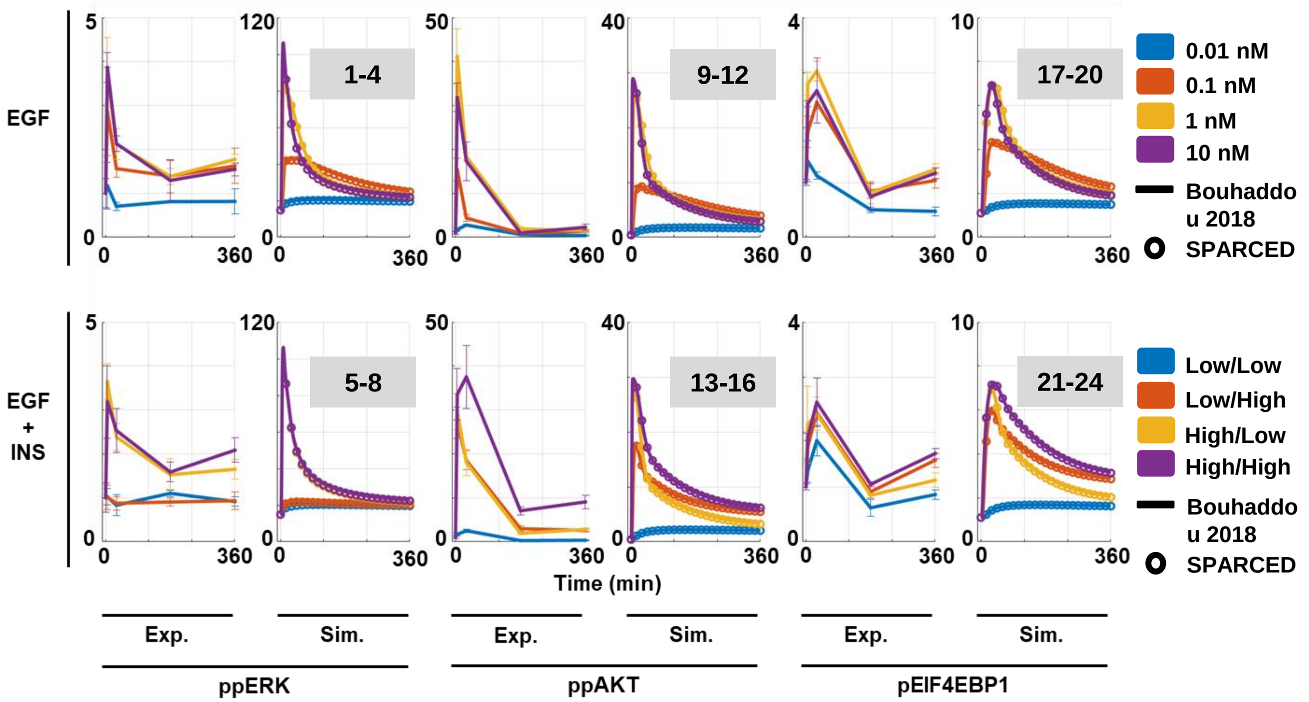

Supplementary Fig. 12 SPARCED model recapitulates downstream pathway activation by ligands and ligand combination treatments. Experimental data and simulation results from MATLAB (lines) and SPARCED (circles) models with EGF (top) and EGF+Insulin (bottom) stimulation for 6 hours. Plots show double-phosphorylated ERK (ppERK), serine-phosphorylated AKT (pAKT), and phospho-EIF4EBP1 (pEIF4EBP1) levels. The numbers in gray shaded boxes represents numbering of conditions in Fig. 3a. Exp: Experimental data, Sim: Simulation. Source data are provided in Source Data.

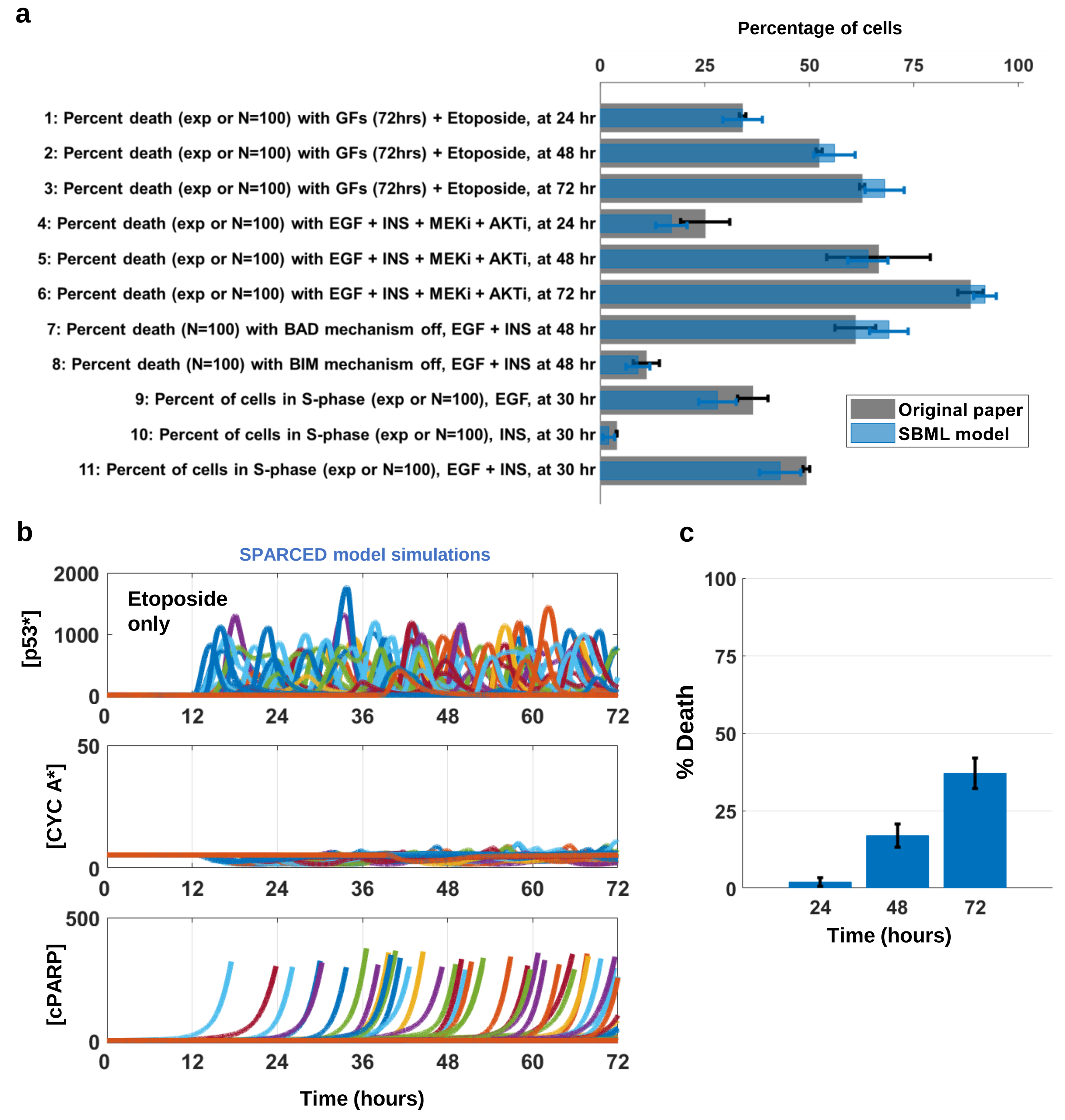

Supplementary Fig. 13 SPARCED simulations recapitulates BOuhaddou2018 model simulations and experimental observations. a Bar plots corresponding to the conditions shown in Fig. 3c. Gray bars are experimental or simulation data from Bouhaddou 2018 model and blue bars are simulation results of SPARCED model. Bars represent mean ± s.e.m. **b** Etoposide treatment alone induces lesser cell death compared to Etoposide + Growth Factor stimulation, shown in Fig. 3d-e. **c** Percentage of cell death of N=100 cells shown in **(b)**. Bars represent percent death ± s.e.m. Source data are provided in Source Data.

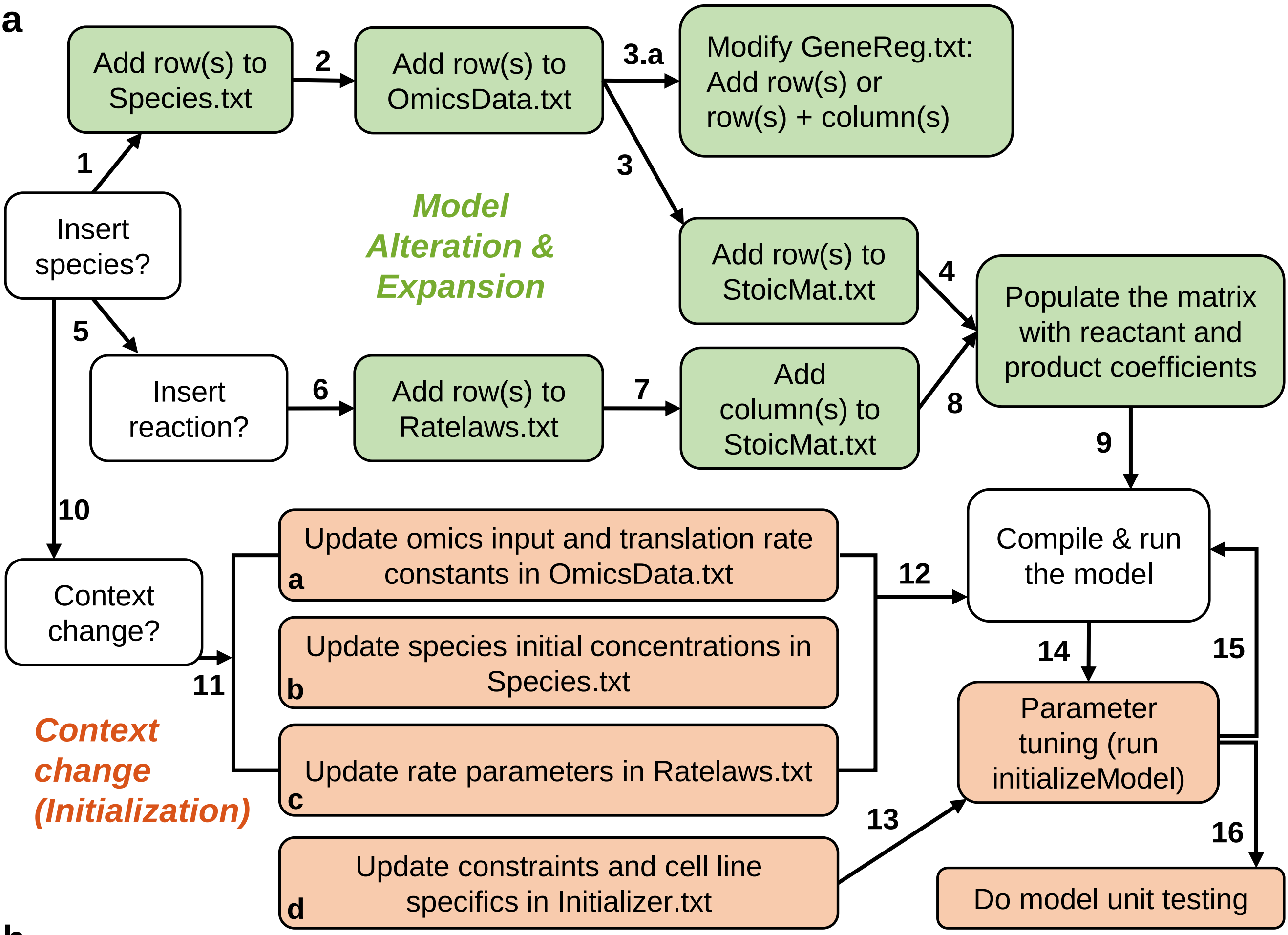

b

Parameter category changed	Number of parameters changed (out of 3275 possible)	Input file
mRNA counts	141	OmicsData.txt
Gene copy numbers	141	OmicsData.txt
Constitutive mRNA translation rates	141	OmicsData.txt
Induced mRNA translation rates (from Bouhaddou2018)	141	Ratelaws.txt
Degradation rate constants (from Bouhaddou2018)	46	Ratelaws.txt
Species initial concentrations (from Bouhaddou2018)	914 (out of 914)	Species.txt

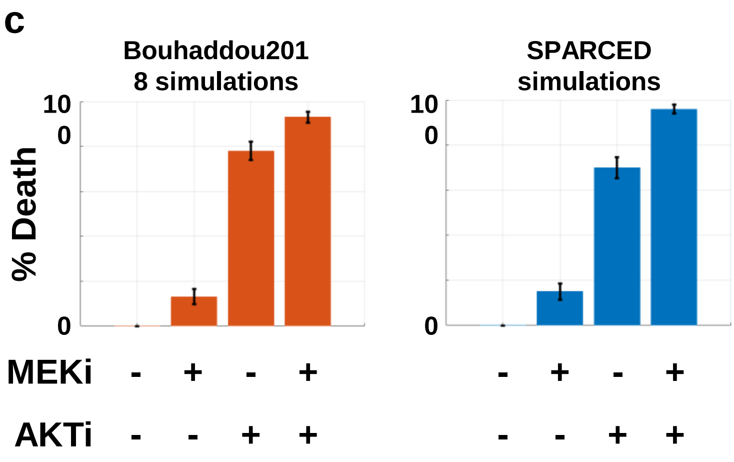

Supplementary Fig. 14 SPARCED model alteration guidelines. a Steps of model expansion and context change procedures are listed. Refer to Supplementary File 13 for more details. Steps can be skipped if no changes are necessary. **b** The list of parameters and species values modified for SPARCED model context change from MCF10A cells to U87 cells. **c** SPARCED_U87 model simulations reproduce previous observations, where U87 cells show increased response and sensitivity to AKT inhibition. MEKi: MEK inhibitor, AKTi: AKT inhibitor. Bars represent percent death ± s.e.m. for N=100 single cell simulations for each condition. Source data are provided in Source Data.

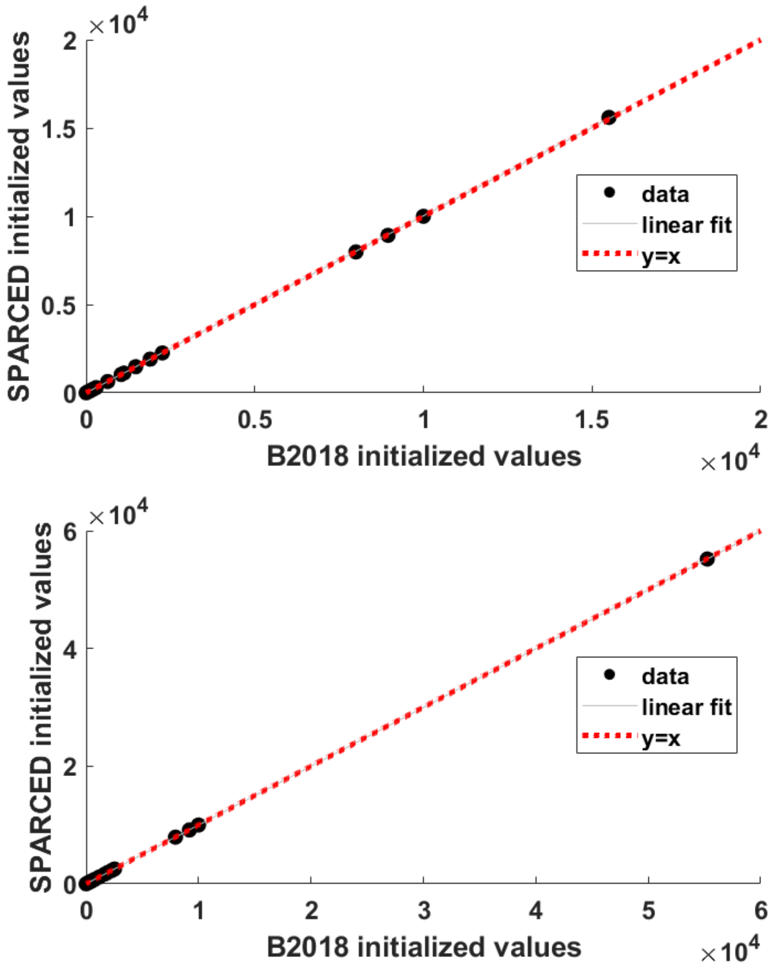

Supplementary Fig. 15 Comparison of initialized species concentrations for MCF10A (top) and U87 (bottom) cells. The Bouhaddou2018 model values are reproduced by the new initialization notebook. The concentrations values (black dots) are almost exact and identity line (dashed red) coincides with the linear fit line (solid black). SPARCED initialized values are on y-axis and Bouhaddou2018 initialized values are on x-axis.

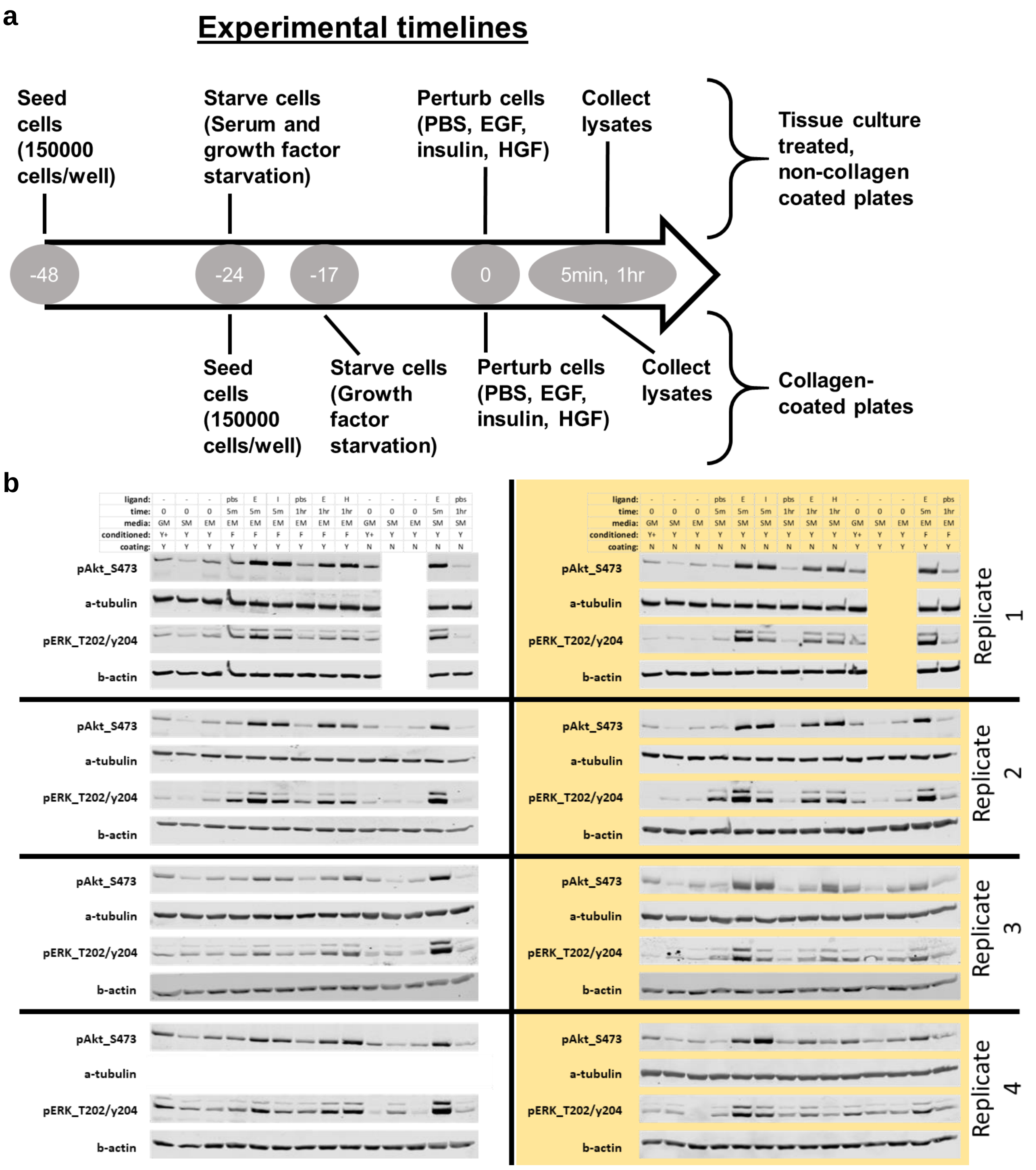

Supplementary Fig. 16 The experimental setup and results of exploring the effect of collagen-coating and serum starvation in MCF10A cells. a The two experimental protocols are followed as shown. **b** Western blot results show elevated AKT response in cells grown on collagen-coated plates compared to non-coated plates. ERK response is not significantly affected.

a

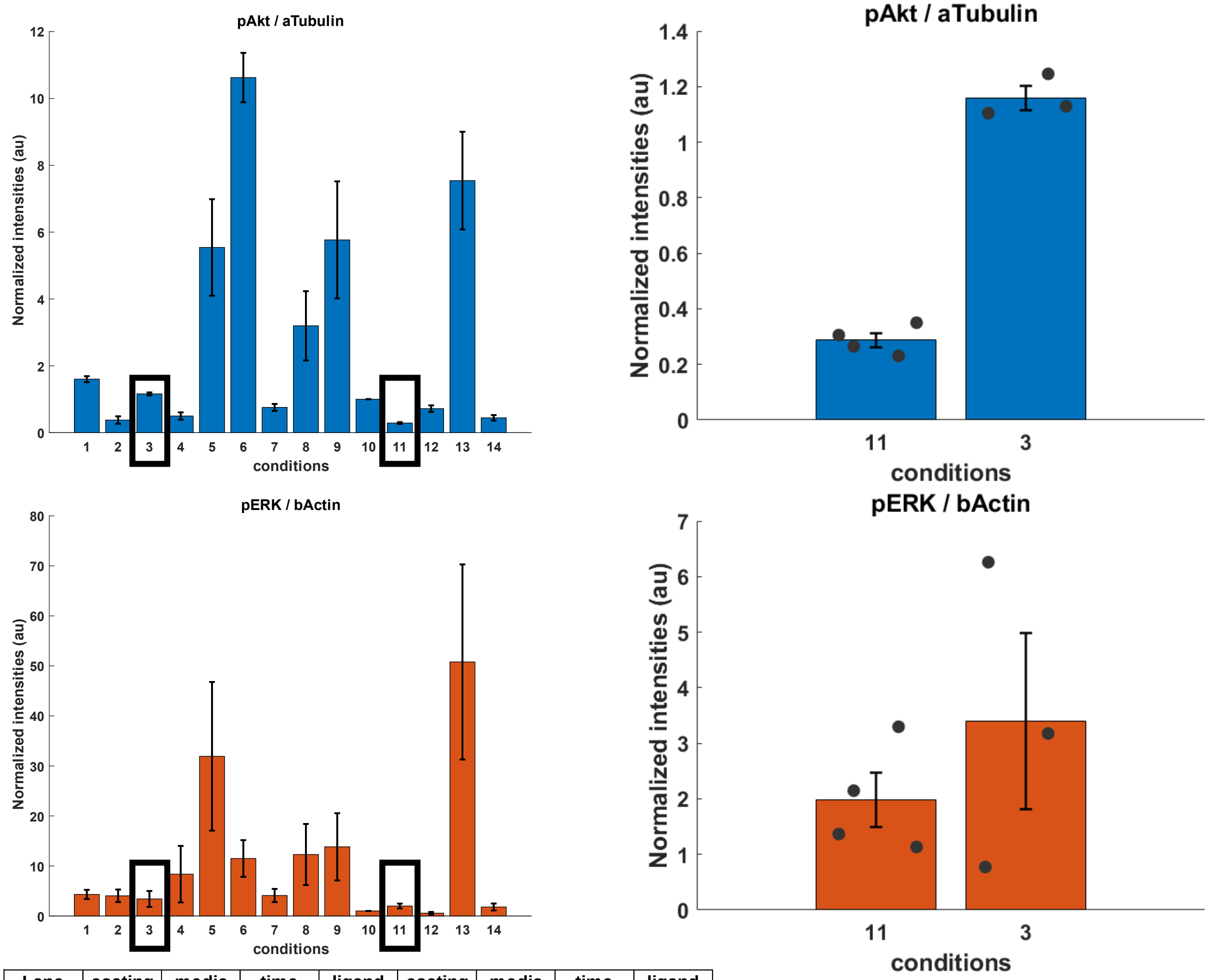

b

Lane	coating	media	time	ligand	coating	media	time	ligand
**1**	Y	GM	0	-	N	GM	0	-
**2**	Y	SM	0	-	N	SM	0	-
**3**	Y	EM	0	-	N	EM	0	-
**4**	Y	EM	5m	PBS	N	SM	5m	PBS
**5**	Y	EM	5m	EGF	N	SM	5m	EGF
**6**	Y	EM	5m	INS	N	SM	5m	INS
**7**	Y	EM	1hr	PBS	N	SM	1hr	PBS
**8**	Y	EM	1hr	EGF	N	SM	1hr	EGF
**9**	Y	EM	1hr	HGF	N	SM	1hr	HGF
**10**	N	GM	0	-	Y	GM	0	-
**11**	N	SM	0	-	Y	SM	0	-
**12**	N	EM	0	-	Y	EM	0	-
**13**	N	SM	5m	EGF	Y	EM	5m	EGF
**14**	N	SM	1hr	PBS	Y	EM	1hr	PBS

Supplementary Fig. 17 Quantification of Western blot images. a Data are normalized to condition 10 (cells in non-coated plates with full growth media, measurements at time 0 hr). pAKT levels in growth factor starved cells in collagen-coated plates (#3) are four times higher than pAKT levels in serum+growth factor starved cells in non-coated plates (#11) (p-value = 8.92E-06). No significant change in pERK levels between two conditions (#3 and #11, p-value = 0.37). Results shown are from four and three biological replicates (dots on bar plots) for conditions 11 and 3, respectively. Bars represent mean ± s.e.m. The order of bars are given according to the list on the left in panel **(b)**. **b** The experimental conditions of western blots membranes in Supplementary Figs. 16, 18, and 19. The raw and normalized data are given in Supplementary File 24. Source data are provided in Source Data.

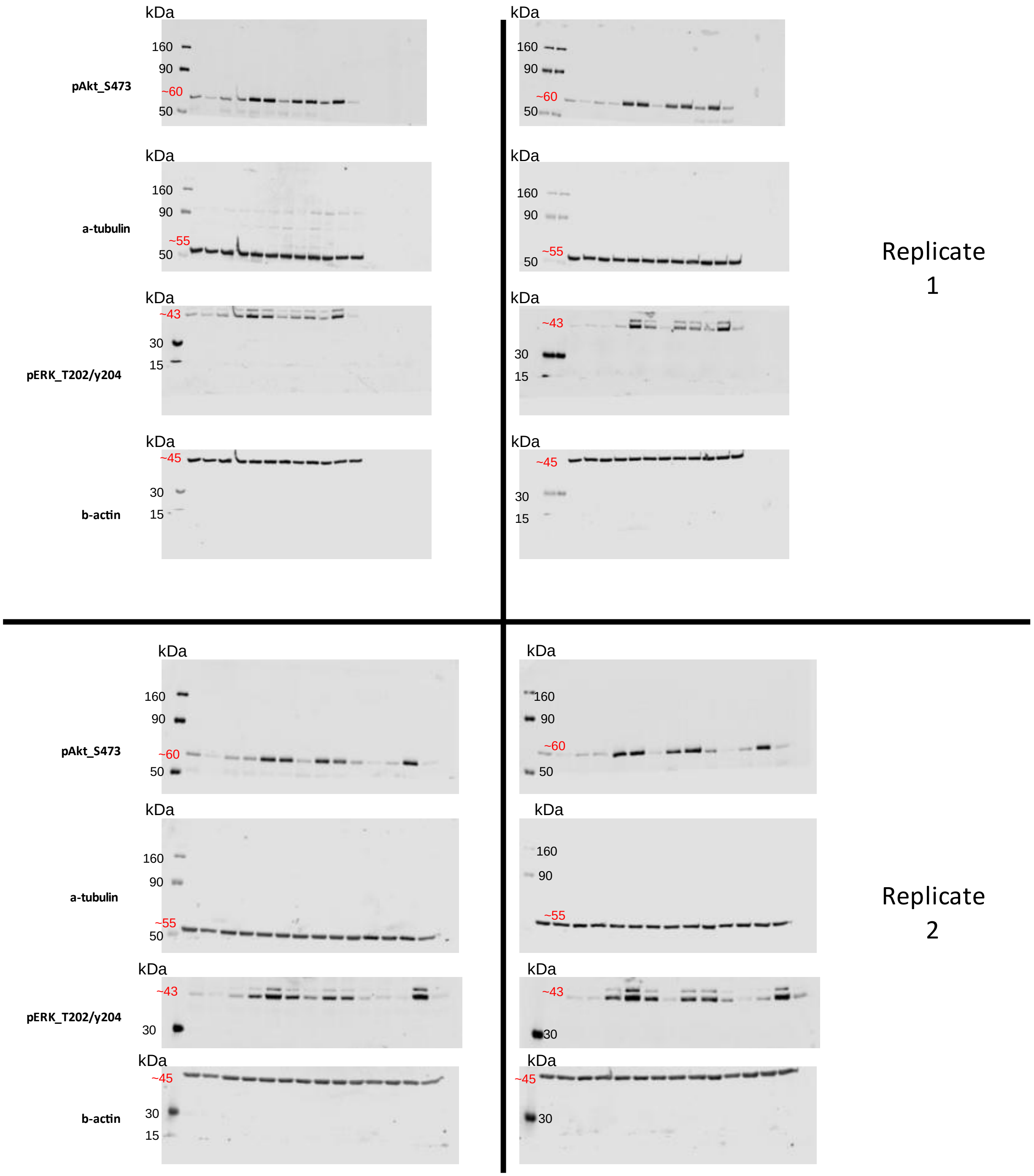

Supplementary Fig. 18 The full images of western blots (replicates 1 and 2) shown in Supplementary Fig. 16.

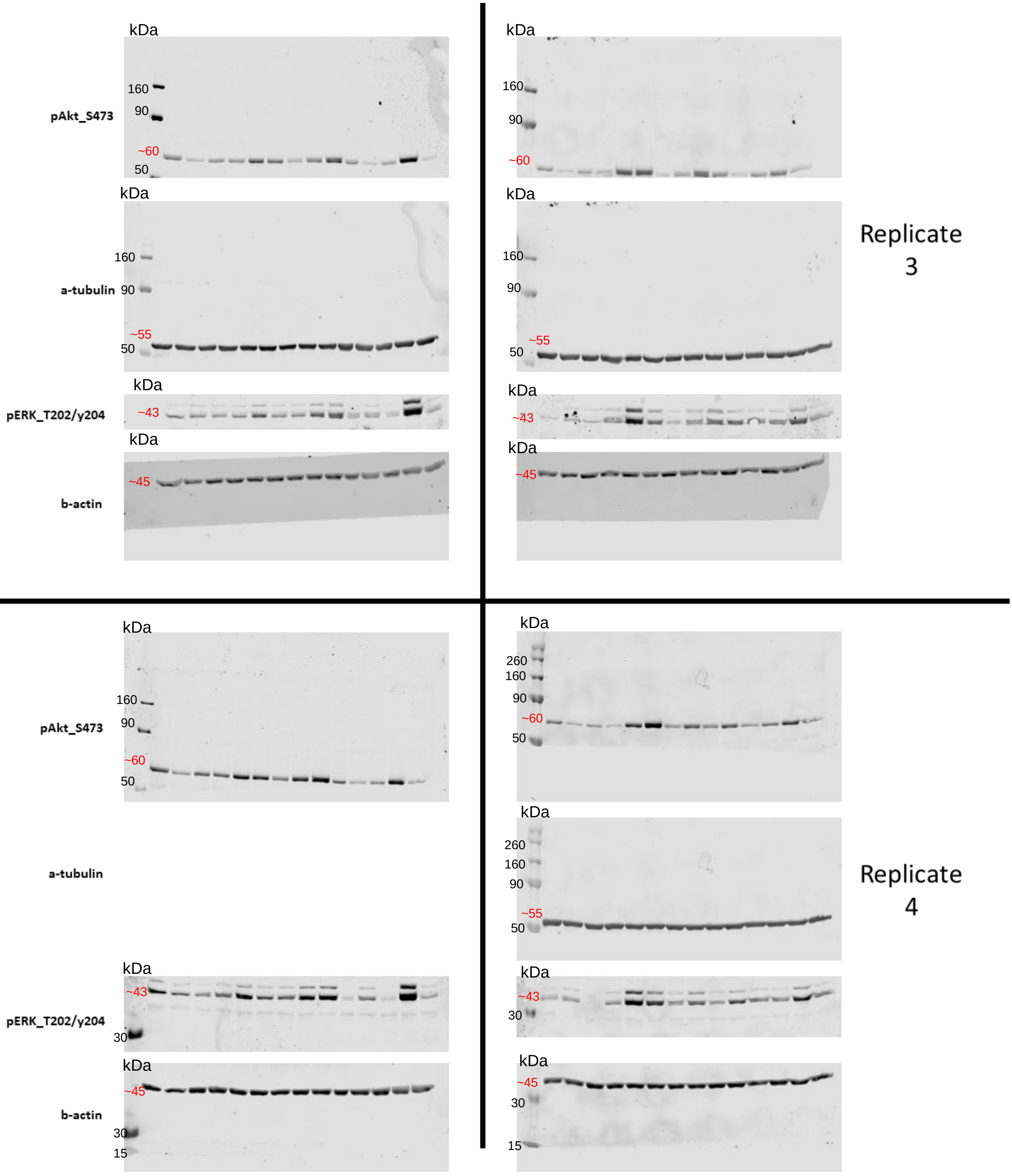

Supplementary Fig. 19 The full images of western blots (replicates 3 and 4) shown in Supplementary Fig. 16.

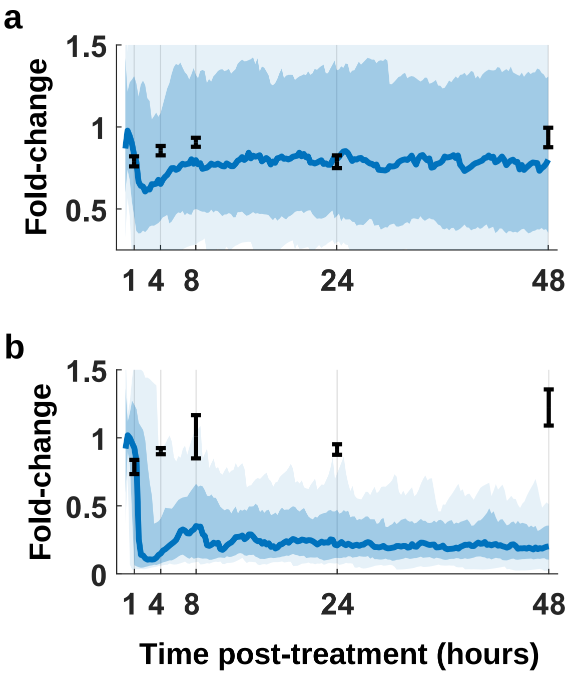

Supplementary Fig. 20 SOCS1 binding/unbinding rates affect cell proliferation. a Normalized pMAPK levels show a significant change when IFNγ is included in addition to the EGF. **b** Normalized ppAKT levels show a significant decrease after IFNγ treatment, when EGF+IFNγ simulations are compared to EGF alone case. RPPA data are shown in black, from three independent replicates. Colored dark lines represent median cell trajectories from simulations, dark and light-colored regions represent 70th and 95th quantiles, respectively. Simulations are from N=100 starting stochastic cells (K_d=1 condition) and experimental data are accessible at synapse.org/LINCS_MCF10A. Source data are provided in Source Data.
