# Supplementary material for: A scalable, open-source implementation of a large-scale mechanistic model for single cell proliferation and death signaling
Source: Nat Commun. 2022 Jun 21;13:3555. doi: 10.1038/s41467-022-31138-1 (PMC9213456; doi:10.1038/s41467-022-31138-1)
Supplement: Supplementary file 2 — Editorial Assessment Report [file 41467_2022_31138_MOESM2_ESM.pdf]

Contents of this report

1. **Manuscript details**: overview of your manuscript and the editorial team.
2. **Review synthesis**: summary of the reviewer reports provided by the editors.
3. **Editorial recommendation**: personalized evaluation and recommendation from all 3 journals.
4. **Annotated reviewer comments**: the referee reports with comments from the editors.
5. **Open research evaluation**: advice for adhering to best reproducibility practices.

About the editorial process

Because you selected the **Nature Portfolio Guided Open Access** option, your manuscript was assessed for suitability in three of our titles publishing high-quality work in your field of research. More information about Guided Open Access can be found here.

Collaborative editorial assessment

Your editorial team discussed the manuscript to determine its suitability for the Nature Portfolio Guided OA pilot. Our assessment of your manuscript takes into account several factors, including whether the work meets the technical standard of the Nature Portfolio and whether the findings are of immediate significance to the readership of at least one of the participating journals in the Guided OA pilot.

Peer review

Experts were asked to evaluate the following aspects of your manuscript:

- **Novelty** in comparison to prior publications;
- **Likely audience** of researchers in terms of broad fields of study and size;
- **Potential impact** of the study on the immediate or wider research field;
- **Evidence** for the claims and whether additional experiments or analyses could feasibly strengthen the evidence;
- **Methodological detail** and whether the manuscript is reproducible as written;
- Appropriateness of the **literature review**.

Editorial evaluation of reviews

Your editorial team discussed the potential suitability of your manuscript for each of the participating journals. They then discussed the revisions necessary in order for the work to be published, keeping each journal's specific editorial criteria in mind.

Journals in the Nature portfolio will support authors wishing to transfer their reviews and (where reviewers agree) the reviewers' identities to journals outside of Springer Nature.
If you have any questions about review portability, please contact our editorial office at guidedoa@nature.com.

nature portfolio GUIDED OPEN ACCESS

Manuscript details

Tracking number	Submission date	Decision date	Peer review type
GUIDEDOA-21-00191	**Jul 16, 2021**	**Oct 27, 2021**	Single-blind

Manuscript title

A Scalable, Open-Source Implementation of a Large-Scale Mechanistic Model for Single Cell Proliferation and Death Signaling

Preprint: A preprint is available at bioRxiv.

Author details

Marc Birtwistle

Affiliation: Clemson University

Editorial assessment team

**Primary editor**	**Ross Cloney**<br>**Home journal:** *Nature Communications*<br>**ORCID:** 0000-0003-3415-5468<br>**Email:** ross.cloney@nature.com
**Other editors consulted**	**Jie Wang,** *Nature Cell Biology*, **ORCID:** 0000-0001-7797-6472<br><br>**Anam Akhtar,** *Communications Biology*, **ORCID:** 0000-0002-8820-8468
**About your primary editor**	Ross joined *Nature Communications* in November 2014. Like many synthetic biologists, he came to the field from other areas of molecular biology – in his case from an academic background in DNA damage and repair at the University of Sussex. His manuscript remit includes synthetic biology and genome engineering, as well as therapeutic biotechnology. Ross is based in the London office.

Editorial assessment and review synthesis

**Editor's summary and assessment**	The authors had previously published in reference 40 a mechanistic model to predict how synergistic mitogen or drug combinations that alter cancer drive pathways affect cancer cell proliferation and death. In this work they use SBML (Systems Biology Markup Language) to convert the previous model into an open-source format called SPARCED, which can be expanded through the incorporation of additional modules.<br><br>Key benefits to the community are the open-source nature and simplified input files to allow easier model alternation and data integration.
**Editorial synthesis of reviewer reports**	While the reviewers note the benefit of a more accessible model, there are significant concerns regarding the technical advance this represents. In addition, reviewers note a mismatch between model predictions and experimental data, inability to capture heterogeneity in a cellular population, and uncertainty regarding assumptions underlying the parameters used.

Editorial recommendation

Nature Cell Biology<br><br>Revision not invited	Both the editors at this journal and reviewers #1 and #2 have raised concerns regarding the incremental technical advance over other approaches and lack of biological insights. These concerns have precluded further consideration at this journal.
Nature Communications<br><br>Major revisions with extension of the work	For further consideration at *Nature Communications*, all technical concerns must be fully addressed. In addition, the work would need to be further developed along the lines indicated by Reviewer #1, including demonstration of superiority to other approaches as well as being able to capture heterogeneity in cell populations.
Communications Biology<br><br>Major revisions	For consideration at *Communications Biology*, we also ask for all technical concerns to be addressed as pointed out by the reviewers, but we are happy to forgo the request for demonstration of a biological insight or conclusion.

Next steps

**Editorial recommendation 1:**	Our top recommendation is to revise and resubmit your manuscript to *Communications Biology.* This option might be best if the requested experimental revisions are not possible/feasible at this time.
**Editorial recommendation 2:**	You may also choose to revise and resubmit your manuscript to *Nature Communications.* We feel the additional experiments required are reasonable.
**Note**	As stated on the previous page *Nature Cell Biology* is not inviting a revision at this time. Please keep in mind that the journal will not be able to consider any appeals of their decision through Guided Open Access.

Revision

To follow our recommendation, please upload the revised manuscript files using **the link provided in the decision letter**. Should you need assistance with our manuscript tracking system, please contact Adam Lipkin, our Nature Portfolio Guided OA support specialist, at guidedOA@nature.com.

Revision checklist

- ☐ Cover letter, stating to which journal you are submitting
- ☐ Revised manuscript
- ☐ Point-by-point response to reviews
- ☐ Updated Reporting Summary and Editorial Policy Checklist
- ☐ Supplementary materials (if applicable)

Submission elsewhere

If you choose not to follow our recommendations, you can still take the reviewer reports with you.

Option 1: Transfer to another Nature Portfolio journal
Springer Nature provides authors with the ability to transfer a manuscript within the Nature Portfolio, without the author having to upload the manuscript data again. To use this service, **please follow the transfer link provided in the decision letter.** If no link was provided, please contact guidedOA@nature.com.

> *Note that any decision to opt in to In Review at the original journal is not sent to the receiving journal on transfer. You can opt in to In Review at receiving journals that support this service by choosing to modify your manuscript on transfer.*

Option 2: Portable Peer Review option for submission to a journal outside of Nature Portfolio
If you choose to submit your revised manuscript to a journal at another publisher, we can share the reviews with another journal outside of the Nature Portfolio if requested. You will need to request that the receiving journal office contacts us at guidedOA@nature.com. We have included editorial guidance below in the reviewer reports and open research evaluation to aid in revising the manuscript for publication elsewhere.

Annotated reviewer reports

The editors have included some additional comments on specific points raised by the reviewers below, to clarify requirements for publication in the recommended journal(s). However, please note that all points should be addressed in a revision, even if an editor has not specifically commented on them.

Reviewer #1 information	
**Expertise**	Mathematical models of cell signalling
**Editor's comments**	While the Reviewer appreciated the development of a more user-friendly approach, they raised severe concerns regarding implementation, advance, insight and the assumptions underpinning the parameters used.

Reviewer #1 comments	
**Section**	**Annotated Reviewer Comments**
**Remarks to the Author: Overall significance**	This paper presents a large-scale model of a mammalian cell that incorporates multiple signaling pathways including growth factor signaling that drives proliferation/growth, MAP kinase, apoptosis, the cell cycle, dna damage, and transcription/translation of the various proteins that make up the networks driving these processes. This model was previously developed by the Birtwistle lab and published in 2018 in PLOS Computational Biology (Bouhaddou2018). The 2018 version of the model was written in MATLAB, and although the code was made freely available upon publication its complexity has made it difficult for others to replicate the original results or to extend the model. The current manuscript describes the group's effort to develop a more user-friendly and extensible version that can serve as the basis for broader development of whole-cell mammalian signaling models. The new version of the model has a more streamlined input in the form of tabular text files that specify each of the model components, including genes, cell compartments, and the species and reactions that make up the signaling network. This model specification is then processed either interactively using a series of scripts embedded in Jupyter notebooks or using a workflow using the Nextflow package, which enables resource-intensive analyses such as parameter scans or simulations of large numbers of cells to be performed on a type of distributed computing resource that is widely available in the academic community. Two case studies demonstrate how the platform can be used to model different cell types by modifying omics data input, and can be used to incorporate an additional signaling pathway and then analyzed to address mechanistic questions.
**Remarks to the Author: Impact**	Although I feel the modeling pipeline presented here does have some value, I think that overall the weaknesses of the manuscript and associated pipeline

	outweigh the strengths. Like its predecessor, the Bouhaddou2018 model, the general impression that one comes away with about this implementation is that it is ad hoc and clunky, and though it represents an ambitious undertaking and has some good ideas behind it, it is unlikely to be of use to the broader community in its current form. I think it is more likely that people who want to build models of this scope may borrow aspects of the design and adapt them to their own styles and preferences. In that sense, I view this paper more as a recipe for how to construct a pipeline than a paper about a pipeline that others will use. I think the paper might elicit a more favorable reaction from the community if it presented itself more in that spirit than it currently does.
**Remarks to the Author: Strength of the claims**	I don't think it is necessary for the authors to address all of the weaknesses that can be identified in the pipeline as it's presented, but I do think they should be more careful in the claims they are making about its advantages and do more to acknowledge its shortcomings. I think the level of general interest in this work is likely to be modest, because what is presented is largely just a refactoring of a mammalian cell model that was previously published in 2018. A sizable part of the presentation here is devoted to demonstrating that the current model gives identical results to the previous one, which, although important, does not substantially extend the state-of-the-art. The authors do not even claim, for instance, that the refactored model gives improved performance in terms of time or computational resources needed to perform a given modeling task. The case studies are presented to demonstrate the greater flexibility of the model, but do not provide any novel biological insights. In fact, I have serious reservations about the way in which the second case study is performed because it neglects the effects of model parameter uncertainty. I have divided the remainder of my critique into major and minor points for the authors to consider in a revision. I don't think all of these need to be resolved in order to publish the paper, but I think addressing them even partially would raise the significance of the work for the biological modeling community.<br><br>**Major points**<br><br>1. Format of primary input files, particularly the input of the reaction network as a stoichiometry matrix does not lend itself to model transparency or model extension, and seems prone to error. It would make more sense to specify the network using a standard reaction format, which would be much easier to read and extend, and would also be more amenable to annotation in the form of references, module names, or formal identifiers. Having a "simple set of structured and annotated input files" (l. 148) is touted as one of the major features of the restructured pipeline, but the reaction input is a text file that has hundreds of rows (species) and thousands of columns. The input file for the basic model (from Bouhaddou2018) is 4.5 Mb and causes my text editor to hang, so the only effective way that I could find to edit it was to open it in Excel. So while the authors claim that switching from

Excel-based input to text-based input is one of the advantages of the new framework, in fact to modify the reaction network you basically need to use a spreadsheet editor. The use of the stoichiometry matrix for input makes it much harder to modify the reactions that make up the core modules of the signaling network, because to undertake a modification one has to first understand what reactions are present. Doing so inside of a gigantic spreadsheet would be almost impossible because the data for each reaction is very sparse (most entries are zero). The more obvious approach would be to convert the stoichiometry matrix to Antimony format, where at least the species involved in each reaction are easy to find. Then the user could figure out which species to add and which reactions to modify. In the current setup the user would then be left with the task of mapping those changes back into the stoichiometry matrix in order to generate a new Antimony file. The user would also have to separately modify the rateLaw file. This would be time-consuming and error-prone. These issues could be avoided by using a list of reactions to specify the reaction network input (including rateLaws), probably using the Antimony reaction syntax. Using a tabular format for this would be natural and lend itself to further annotation of the reactions as suggested above. Making this change would make the model much easier to understand and modify.

Editor's note: We agree with the reviewer's request to specify the network using a standard reaction format. Kindly modify your model employing Antimony reaction syntax as suggested by the reviewer here for consideration at *Communications Biology*.

2. Standalone Python implementation of the gene expression module. One of the novel features of the Bouhaddou2018 model was the presentation of a hybrid deterministic-stochastic algorithm for simulating the integrated dynamics of signaling and gene expression. Because of the computational expense of simulating the large number of reactions and protein copy numbers in the signaling network stochastically, the full network was divided into gene expression and signaling components, with gene expression simulated stochastically using an approximate method (essentially Tau-leaping) and the signaling network simulated with ODE's. By integrating the gene expression network into a Python simulation code instead of exporting the gene expression network as a set of reactions in SBML format, this pipeline makes it more difficult to simulate the network using different approaches. The approach taken by Bouhaddou2018 has so far not been studied extensively and its accuracy for modeling the heterogeneity in cell populations is not well established, so it would be desirable to apply different simulation approaches including for example: simulation of the entire network using SSA, simulation of the entire network using ODE's, simulation of the two subnetworks using various hybrid approaches that have been suggested in the literature (e.g.,

https://bmcsystbiol.biomedcentral.com/articles/10.1186/1752-0509-3-89). I think this could be facilitated by enabling the export of 1 or 2 SBML models respectively containing either the full combined reaction network or the network split into gene regulation and signaling modules.

Editor's note: Kindly demonstrate the accuracy for modeling the heterogeneity in cell populations using one of the approaches mentioned here for consideration at *Communications Biology*.

A third option could take advantage of the SBML comp package, which allows for the specification of a model comprised of modules that are linked together via ports. Whether or not any specific new capabilities are added to the current pipeline, I think the current structure should be mentioned as a shortcoming. The current discussion of hybrid simulation in the paragraph beginning at l. 605 could be expanded to address these possibilities. The authors seem to be suggesting there that their approach is superior to other hybrid methods, such as the one implemented in COPASI (l. 611-2) but they do not provide any data to back this up. They also have not demonstrated that their approach accurately captures the heterogeneity in cell populations.

Editor's note: Since the superiority of this approach over other hybrid methods is not established, for *Communications Biology*, at the very least, it is essential to discuss this point as a limitation.

3. The case study on IFNg effects does not acknowledge or take into account the effect of parameter uncertainty. It ends up making a probabilistic statement (mechanism 2 is more likely than mechanism 1) without attempting to quantify any probabilities. The authors do acknowledge that the parameters governing both mechanisms are uncertain, and in fact in Figs. 5D,E where the effects of each on proliferation are compared, both mechanisms demonstrate a reduction in proliferation although the one observed for mech 1 fails to meet a significance test, but this is just for one fairly arbitrarily set of parameters. A rigorous analysis would have to take this uncertainty into account. I don't think the analysis provides sufficient basis for the conclusions that are drawn, including those in lines 44-47 of the abstract.

   Editor's note: We agree that this is an important point and ask that this comment is addressed in full for further consideration at *Communications Biology*.

4. The Discussion should more fully acknowledge the shortcomings and limitations of the current approaches. Some of these have been mentioned above. An additional limitation that is important in the context of multicellular and spatial simulations, as discussed on lines 621-633 is that the model does not consider cell growth and division, nor the closely-related aspect of metabolism. Another limitation is that to this point there has really been no analysis of the predicted vs. observed heterogeneity in single-cell data. Does the model accurately capture the

	distributions that are observed experimentally? Another limitation is that the authors have not followed the recently-developed OMEX standard (doi: 10.1515/JIB-2020-0020) for model distribution and annotation.<br>**Editor's note: (*Communications Biology)* We fully agree with the reviewer's comments and ask that you please expand the discussion as requested.**<br><br>Minor<br>1. Lines 316ff. Unclear that the data being discussed was previously published in Bouhaddou2018.<br>2. Is the experimental data used in the case study of IFNg (Figs. 5F,G) published for the first time here, or was the data previously published?<br><br>**For *Nature Communications*, all points raised must be fully addressed and the work extended as the reviewer suggests.**
**Remarks to the Author: Reproducibility**	The main comment I have related to reproducibility is point 3 above concerning the failure of the case study to take into account model uncertainty. Without a probabilistic analysis, I don't think the authors can draw any conclusions about which mechanism is more likely. The authors seem to reach the conclusion that mechanism 2 is more likely largely on the basis of their interpretation of the experimental data - in particular the observation the Akt is reduced substantially during the first 24 hours after stimulation but p21 is not. It's not clear how the model informs that analysis.<br>**For further consideration at *Communications Biology* we would not strictly require new experimental data, but at a minimum the conclusions should be toned down or modified to more accurately reflect the data.**<br><br>**For *Nature Communications*, all points raised must be fully addressed and the work extended as the reviewer suggests.**

Reviewer #2 information

**Expertise**	Computational medicine and mechanistic modelling
**Editor's comments**	While the reviewer appreciated the development of an open-source model, they raised severe concerns regarding mismatches between model predictions and experimental data.

Reviewer #2 comments

Section	Annotated Reviewer Comments
**Remarks to the Author: Overall significance**	"A Scalable, Open-Source Implementation of a Large-Scale Mechanistic Model for Single Cell Proliferation and Death Signaling" by Erdem et al<br><br>This paper describes a new, open-source, HPC-ready code version of a large-scale model previously published by the authors. It includes an RTK module, signaling and cell behavior modules. The now open-source model recapitulates previously published observations.<br><br>The paper also illustrates by example how the model can be expanded to incorporate a new module; in this case, IFNgamma (which itself may yield biological insights). The IFNgamma module added is based on a previously published model, though tuning and optimization of some of the relevant parameters was needed to make it interoperable as part of the larger model.<br><br>Thus, the paper could be viewed as a software method introduction and description, and as a new research paper. Its main purpose and its strength is the former; this is enhanced and supported by the incorporation of the model expansion and new simulations, though the biological insight/conclusions I found to be somewhat weak, since there appear to be some mismatches between the model and experimental data. It would be very difficult for any model with so many moving parts to match all data, of course, but the authors don't seem to explore reasons for the mismatches. Overall, since I believe the focus and strength of the paper to be its methodological contributions, and since these will be useful not only to the authors but also provide a framework that others can use and build upon, therefore I don't think these shortcomings in the modeling results section should substantially stand in the way of its publication.<br><br>I found the paper to be generally well-written and although it packs a lot in, I found it easy to read and not over-written. The visualizations are well put together and clear. It's very nice work!

**Remarks to the Author: Impact**	The main impact of this paper is likely to be via the adoption and use of the open source code and methodology presented. It should make the field of modeling (particularly this system, but it can be easily generalized) more accessible. I can see the platform being used in both academic and industry labs, and as a teaching tool.
**Remarks to the Author: Strength of the claims**	A couple of critiques/suggestions:<br><br>1. I'm a bit confused by the IFNgamma mechanism section. Mechanism 1 (STAT1 -> p21) is introduced (p.22) and regulation of p21 transcription by activated STAT1 is added to the model. A rate is assumed, and then simulation results presented that suggest p21 levels don't change substantially with the addition of IFNgamma. On p.25, it's noted that for the first 24 hrs, experimentally p21 doesn't increase but then it does at 48 hours. Despite this mismatch between the experiments and model, the authors state that "We conclude that the original parameter choices are appropriate and that the putative p21 mechanism is therefore unlikely." - I just don't follow how this is supported by the graphs/data presented. Of note here is that Fig S20 suggests that altering the p21 parameter will change the value of Fig 5D, so further exploration of simulations that match the expression experiments may be necessary. In addition, it would help to state more clearly what the expected or observed experimental result is for Fig 5D/E. For mechanism 2, again the 48 hour experimental timepoint appears not to match simulations. It's not obvious, based on the presented results, why mechanism 2 is viewed as possible while mechanism 1 is not. This section ends somewhat abruptly, and feels underdeveloped. It works well as an example of the methodology to expand the model, but (hopefully without huge amounts of additional work) I think can be more clearly explored. As noted elsewhere, I am not advocating for significant amounts of work to exhaustively explore these results because I think the model and methodology itself is highly impactful; at the same time, the inclusion of this example of model expansion is a major part of explaining and illustrating the methodology, so I like that it's included.<br>**Editor's note: We feel this is a very important point raised by the reviewer. Kindly address this mismatch observed satisfactorily for further consideration at *Communications Biology*.**<br><br>2. I think you could be clearer (throughout the manuscript) in describing what you mean by 'single-cell' - one cell per simulation, many distinct cells per simulation, etc.<br><br>3. It's good that description is added throughout the paper as to how to expand/modify the model, as well as the explicit supplemental worksheet on model expansion and model modification (supplement 13; though this is labeled Table 2 in the supplement in the caption of

	Fig S14). A very minor note here is that the numbering of these steps is a little inconsistent/confusing, e.g. 3.1 vs. 11a-d; I get that these numbers match up to the workflow in Fig S14a.<br>4. This is another very minor point, and likely beyond the control of the authors, but the vagaries of the Nature filename system mean that the supplemental code files don't download with the correct names. While most people will (should?) get the files from the GitHub repository, it may make sense to include in the supplement or elsewhere some guide to the headers of each file so that they can be easily identified should the reader get them from the supplemental file source. The materials & methods section does this somewhat.<br><br>**For *Nature Communications*, all points raised must be fully addressed.**
**Remarks to the Author: Reproducibility**	Good visualizations, statistical analysis seems fine, and the availability of the code (as well as the instructions on how to modify it) should make for good reproducibility.

Reviewer #3 information

**Expertise**	Mechanistic modelling, Single Cell Data Analysis
**Editor's comments**	This reviewer appreciated the development of an open-source model.

Reviewer #3 comments

Section	Annotated Reviewer Comments
**Remarks to the Author: Overall significance**	The paper presents a thorough methodology and an open-source tool (SPARCED) developed by the authors with the aim to analyze single cell RNASeq studies from a mechanistic and integrative perspective that uses proteomic and transcriptomic data, but focused on cell proliferation and cell death outcomes. Authors use data from LINCS database in order to validate the methodology and to present some interesting results regarding MCF10A, a breast fibrosis derived cell line.
**Remarks to the Author: Impact**	Systems biology and network biology represent the current trends in biomedical studies, nevertheless this approach is still developing and we need new but reliable resources and tools in order to perform mechanistic analyses.

	This paper offers a new methodology and framework (based on their previous work) for mechanistic modeling of cell death and proliferation, both hallmarks of cancer, which prediction would be very useful in cancer management and research, to evaluate both prognosis and therapeutic approaches.
**Remarks to the Author: Strength of the claims**	1. The introduction is beautifully written and covers all aspects of the state of the art and previous tools available, as well as some concepts needed to understand the paper.<br>2. The methodology is thoroughly described, perhaps a little overwhelming, but it is preferable this way.<br>3. The use of stochastic differential equations is well reasoned and supported and the implementation seems to be well made.<br>4. The results are interesting, although authors acknowledge that commenting on the results from the model is not of the scope of the paper, I miss some biological discussion, but this lack in no case diminishes the value of the work.<br>5. To my (limited) knowledge, discussion covers many of the current tools and approaches, and comments the capabilities and improvements of SPARCED, alongside some limitations and comments on future work.<br><br>For all, I support the publication of this manuscript.<br><br>**For *Nature Communications*, all points raised must be fully addressed and the discussion of biological results extended as the reviewer suggests.**
**Remarks to the Author: Reproducibility**	The methodology is carefully and thoroughly explained and all the information needed is of high-quality and available for reproducibility.

Open research evaluation

Data availability

Data availability statement

Thank you for including a Data Availability statement. However, we noted that you have only indicated that data are available upon request. The data availability statement must make the conditions of access to the “minimum dataset” that are necessary to interpret, verify and extend the research in the article, transparent to readers.

In addition, Nature Portfolio policies include a strong preference for research data to be archived in public repositories. For data types without specific repositories, we recommend that data are deposited in a generalist repository such as figshare or Dryad. More information about our data availability policy can be found here: https://www.nature.com/nature-portfolio/editorial-policies/reporting-standards#availability-of-data

See here for more information about formatting your Data Availability Statement: http://www.springernature.com/gp/authors/research-data-policy/data-availability-statements/12330880

Mandatory data deposition

For your RNA sequencing data, submission to a community-endorsed, public repository is mandatory for publication in a Nature Portfolio journal and is best practice for publication in any venue. Accession numbers must be provided in the paper. Examples of appropriate public repositories are listed below:

- Gene Expression Omnibus (Microarray or RNA sequencing data)
- Sequence Read Archive (high-throughput sequence data)
- The European Nucleotide Archive (ENA)

More information on mandatory data deposition policies at the Nature Portfolio can be found at http://www.nature.com/authors/policies/availability.html#data

Please visit https://www.springernature.com/gp/authors/research-data-policy/repositories/12327124 for a list of approved repositories for each mandatory data type.

For your genome-wide association study, submission of the full linked genotype dataset to a community-endorsed, public repository is mandatory for publication in a Nature Portfolio journal and is best practice for publication in any venue. Accession numbers must be provided in the paper.

For this data type, we recommend submission to the NCBI Sequence Read Archive (SRA): https://www.ncbi.nlm.nih.gov/sra

We also strongly encourage you to deposit full summary statistics and other related data to a generalist repository, such as figshare or Dryad. However, it may be acceptable to include the summary statistics in the supplementary information.

More information on mandatory data deposition policies at the Nature Portfolio can be found at http://www.nature.com/authors/policies/availability.html#data

Please visit https://www.springernature.com/gp/authors/research-data-policy/repositories/12327124 for a list of approved repositories for each mandatory data type.

Because your study includes human participants, confirmation that all relevant ethical regulations were followed is needed, and that informed consent was obtained. This must be stated in the Methods section, including the name of the board and institution that approved the study protocol.

Reporting & reproducibility

We recommend reporting as per the Minimal Information for Studies of Extracellular Vesicles 2018 guidelines: https://www.tandfonline.com/doi/full/10.1080/20013078.2018.1535750

Please refer to the MISEV2018 quick reference checklist at the end of the document.

Nature Portfolio journals allow unlimited space for Methods. The Methods must contain sufficient detail such that the work could be repeated. It is preferable that all key methods be included in the main manuscript, rather than in the Supplementary Information.

Please avoid use of “as described previously” or similar, and instead detail the specific methods used with appropriate attribution.

We encourage you to share your step-by-step experimental protocols on a protocol sharing platform of their choice. The Nature Portfolio’s Protocol Exchange is a free-to-use and open resource for protocols; protocols deposited in Protocol Exchange are citable and can be linked from the published article. More details can be found at www.nature.com/protocolexchange/about

Statistics and data presentation

To improve reproducibility of your analyses, please provide details regarding your treatment of outliers.

The quality of some of the figures appears to be quite low. If possible, we suggest replacing these with higher-resolution images.

Data presentation: Please ensure that data presented in a plot, chart or other visual representation format shows data distribution clearly (e.g. dot plots, box-and-whisker plots). When using bar charts, please overlay the corresponding data points (as dot plots) whenever possible and always for n ≤ 10. (Please see the following editorial for the rationale behind this request and an example https://www.nature.com/articles/s41551-017-0079).

Panels requiring revision:
Please note that data presentation has to be revised to comply with our policy in figures 3e, 5d-e, supplementary figures 9a-e, 10a-b, 13c, 14c, 17a, 20b.

Statistics: Wherever statistics have been derived (e.g. error bars, box plots, statistical significance) the legend needs to provide and define the n number (i.e. the sample size used to derive statistics) as a precise value (not a range), using the wording “n=X biologically independent samples/animals/cells/independent experiments/n= X cells examined over Y independent experiments” etc. as applicable.

Legends requiring revision:

- Please note that this information is missing in the legends of figures 3e, 5d-e, supplementary figures 6c, 9a-e, 13c.
- Please provide a precise value of ‘n’ in the legend of supplementary figure 17a.

Please note that statistics such as error bars significance and p values cannot be derived from n<3 and must be removed in all such cases.

We strongly discourage deriving statistics from technical replicates, unless there is a clear scientific justification for why providing this information is important. Conflating technical and biological variability, e.g., by pooling technically replicates samples across independent experiments is strongly discouraged. (For examples of expected description of statistics in figure legends, please see the following https://www.nature.com/articles/s41467-019-11636-5 or https://www.nature.com/articles/s41467-019-11510-4) .

All error bars need to be defined in the legends (e.g. SD, SEM) together with a measure of centre (e.g. mean, median). For example, the legends should state something along the lines of “Data are presented as mean values +/- SEM” as appropriate.
All box plots need to be defined in the legends in terms of minima, maxima, centre, bounds of box and whiskers and percentile.

Legends requiring revision:
Please note that the error bars need to be defined in the legends of figures 5d-e, supplementary figures 9a-e, 17a, 20b.

The figure legends must indicate the statistical test used. Where appropriate, please indicate in the figure legends whether the statistical tests were one-sided or two-sided and whether adjustments were made for multiple comparisons.
For null hypothesis testing, please indicate the test statistic (e.g. F, t, r) with confidence intervals, effect sizes, degrees of freedom and P values noted.
Please provide the test results (e.g. P values) as exact values whenever possible and with confidence intervals noted.

Legends requiring revision:
Please note that the exact p value should be provided, when possible, in the legends of figures 4a, 5e.

Reproducibility: Please state in the legends how many times each experiment was repeated independently with similar results. This is needed for all experiments, but is particularly important wherever results from representative experiments (such as micrographs) are shown. If space in the legends is limiting, this information can be included in a section titled “Statistics and Reproducibility” in the methods section.

Please ensure that datasets deposited in public repositories are now publicly accessible, and that accession codes or DOI are provided in the "Data Availability" section. As long as these datasets are not public, we cannot proceed with the acceptance of your paper. For data that have been obtained from publicly available sources, please provide a URL and the specific data product name in the data availability statement. Data with a DOI should be further cited in the methods reference section.

Gels and Blots: Quantitative comparisons between samples on different gels/blots are discouraged; if this is unavoidable, the figure legend must state that the samples derive from the same experiment and that gels/blots were processed in parallel.
Vertically sliced images that juxtapose lanes that were non-adjacent in the gel must have a clear separation or a black line delineating the boundary between the gels. Loading controls (e.g. GAPDH, actin) must be run on the same blot.
Sample processing controls run on different gels must be identified as such in the figure legends, and distinctly from loading controls.
All blots and gels must be accompanied by the locations of molecular weight/size markers. Blots should be cropped such that at least one marker position is present.
Please also supply uncropped and unprocessed scans of the most important blots in the Source Data file or as a supplementary figure in the Supplementary Information. This should be cited once in the Methods section.For an example of presentation of full scan blots, see the Source Data file of https://www.nature.com/articles/s41467-020-16984-1#Sec35 and for more information, please refer to https://www.nature.com/nature-research/editorial-policies/image-integrity

Panels requiring revision:
Please note that molecular weight markers are missing for supplementary figure 16b.

Language editing

The English language in your text would benefit from improvement for clarity and readability. We recommend that you either ask a colleague with strong English language skills to review your manuscript or that you use one of the many English language editing services available. Two such services are provided by our affiliates:

- Springer Nature Editing Service: https://secure.authorservices.springernature.com/en/researcher/submit/upload
- American Journal Experts: https://www.aje.com/go/natureresearch/

Other notes

We have included as an attachment to the decision letter a version of your Reporting Summary with a few notes. This is mainly for your information, but we hope it is helpful when preparing your revised manuscript. If you decide to resubmit the manuscript for further consideration, please be sure to include an updated Reporting Summary.
