# Supplementary material for: A scalable, open-source implementation of a large-scale mechanistic model for single cell proliferation and death signaling
Source: Nat Commun. 2022 Jun 21;13:3555. doi: 10.1038/s41467-022-31138-1 (PMC9213456; doi:10.1038/s41467-022-31138-1)
Supplement: Supplementary file 3 — Description of Additional Supplementary Files [file 41467_2022_31138_MOESM3_ESM.pdf]

Description of Additional Supplementary Files

File Name: Supplementary Data 1

Description: OmicsData input file

File Name: Supplementary Data 2

Description: Species input file

File Name: Supplementary Data 3

Description: Ratelaws input file

File Name: Supplementary Data 4

Description: Stoichiometric Matrix input file

File Name: Supplementary Data 5

Description: Gene Regulation input file

File Name: Supplementary Data 6

Description: Compartments input file

File Name: Supplementary Data 7

Description: Observables input file

File Name: Supplementary Data 8

Description: Model creation Jupyter notebook

File Name: Supplementary Data 9

Description: SPARCED model Antimony file

File Name: Supplementary Data 10

Description: SPARCED model SBML file

File Name: Supplementary Data 11

Description: Model import and simulation Jupyter notebook

File Name: Supplementary Data 12

Description: Parameter value replacements for U87 cell line SPARCED model

File Name: Supplementary Data 13

Description: SPARCED model alteration steps

File Name: Supplementary Data 14

Description: U87 cell line omics data and model parameter values from the Bouhaddou2018 model

File Name: Supplementary Data 15

Description: Protein-to-mRNA ratios file

File Name: Supplementary Data 16

Description: Model initialization Jupyter notebook

File Name: Supplementary Data 17

Description: Model initialization Jupyter notebook for U87 cell line

File Name: Supplementary Data 18

Description: Initialization input file

File Name: Supplementary Data 19

Description: U87 cell line species input file

File Name: Supplementary Data 20

Description: Gene Regulation input file for SPARCED-I model

File Name: Supplementary Data 21

Description: SPARCED-I model SBML file

File Name: Supplementary Data 22

Description: MCF10A RNA-seq and proteomics data

File Name: Supplementary Data 23

Description: SPARCED-I-SOCS1 model SBML file

File Name: Supplementary Data 24

Description: Raw western blot data
